# On the Non-Adaptive Zero-Error Capacity of the Discrete Memoryless Two-Way Channel †

**DOI:** 10.3390/e23111518

**Published:** 2021-11-15

**Authors:** Yujie Gu, Ofer Shayevitz

**Affiliations:** 1 Faculty of Information Science and Electrical Engineering, Kyushu University, Fukuoka 819-0395, Japan; 2 Department of EE—Systems, Tel Aviv University, Tel Aviv 69978, Israel

**Keywords:** zero-error capacity, two-way channel, Shannon capacity of a graph

## Abstract

We study the problem of communicating over a discrete memoryless two-way channel using non-adaptive schemes, under a zero probability of error criterion. We derive single-letter inner and outer bounds for the zero-error capacity region, based on random coding, linear programming, linear codes, and the asymptotic spectrum of graphs. Among others, we provide a single-letter outer bound based on a combination of Shannon’s vanishing-error capacity region and a two-way analogue of the linear programming bound for point-to-point channels, which, in contrast to the one-way case, is generally better than both. Moreover, we establish an outer bound for the zero-error capacity region of a two-way channel via the asymptotic spectrum of graphs, and show that this bound can be achieved in certain cases.

## 1. Introduction

The problem of reliable communication over a *discrete memoryless two-way channel (DM-TWC)* was originally introduced and investigated by Shannon [1] in a seminal paper that has marked the inception of multi-user information theory. A DM-TWC is characterized by a quadruple of finite input and output alphabets $X_{1}$, $X_{2}$, $Y_{1}$, $Y_{2}$, and a conditional probability distribution $P_{Y_{1},Y_{2}|X_{1},X_{2}}\left(y_{1},y_{2}|x_{1},x_{2}\right)$, where $x_{1}\in X_{1}$, $x_{2}\in X_{2}$, $y_{1}\in Y_{1}$, $y_{2}\in Y_{2}$. The channel is memoryless in the sense that channel uses are independent, that is, for any *i*,

$$\begin{array}{cc} \; & P_{Y_{1i},Y_{2i}|X_{1}^{i},X_{2}^{i},Y_{1}^{i-1},Y_{2}^{i-1}}\left(y_{1i},y_{2i}|x_{1}^{i},x_{2}^{i},y_{1}^{i-1},y_{2}^{i-1}\right)=P_{Y_{1},Y_{2}|X_{1},X_{2}}\left(y_{1i},y_{2i}|x_{1i},x_{2i}\right). \end{array}$$

In [1], Shannon provided inner and outer bounds for the vanishing-error capacity region of the DM-TWC, in the general setting where the users are allowed to adapt their transmissions on the fly based on past observations. We note that Shannon’s inner bound is tight for non-adaptive schemes, namely when the users map their messages to codewords in advance. The non-adaptive DM-TWC is also sometimes called the *restricted* DM-TWC [2]. Shannon’s inner and outer bounds have later been improved by utilizing auxiliary random variable techniques [3,4,5], and sufficient conditions under which his bounds coincide have been obtained [6,7]. However, despite much effort, the capacity region of a general DM-TWC under the vanishing-error criterion remains elusive. In fact, a strong indicator for the inherent difficulty of the problem can be observed in Blackwell’s binary multiplying channel, a simple, deterministic, common-output channel whose capacity remains unknown hitherto [4,5,8,9,10].

In yet another seminal work, Shannon proposed and studied the zero-error capacity of the point-to-point discrete memoryless channel [2], also known as the Shannon capacity of a graph. This problem has been extensively studied by others, most notably in [11,12], yet remains generally unsolved. In this paper, we consider the problem of zero-error communication over a DM-TWC. We limit our discussion to the case of non-adaptive schemes, for which the capacity region is known in the vanishing-error case [1]. Despite the obvious difficulty of the problem (the point-to-point zero-error capacity is a special case), its two-way nature adds a new combinatorial dimension that renders it interesting to study. To the best of our knowledge, this problem has not been addressed before, except in the special case of the binary multiplying channel, where upper and lower bounds on non-adaptive zero-error sum capacity have been obtained [13,14,15]. Our bounds are partially based on generalizations of these ideas and an earlier short version [16].

The problem of non-adaptive communication over a DM-TWC can be formulated as follows. Alice and Bob would like to simultaneously convey messages $m_{1}\in \left[2^{nR_{1}}\right]$ and $m_{2}\in \left[2^{nR_{2}}\right]$ respectively to each other, over *n* uses of the DM-TWC $P_{Y_{1},Y_{2}|X_{1},X_{2}}$. To that end, Alice maps her message to an input sequence (codeword) $x_{1}^{n}\in X_{1}^{n}$ using an encoding function $f_{1}:\left[2^{nR_{1}}\right]\to X_{1}^{n}$, and Bob maps his message into an input sequence (codeword) $x_{2}^{n}\in X_{2}^{n}$ using an encoding function $f_{2}:\left[2^{nR_{2}}\right]\to X_{2}^{n}$. We call the pair of codeword collections $\left(f_{1}\left(\left[2^{nR_{1}}\right]\right),f_{2}\left(\left[2^{nR_{2}}\right]\right)\right)$ a *codebook pair*. Note that the encoding functions depend only on the messages, and not on the observed outputs during the transmission, hence the name *non-adaptive*. When transmissions end, Alice and Bob observe the resulting (random) channel outputs $Y_{1}^{n}\in Y_{1}^{n}$ and $Y_{2}^{n}\in Y_{2}^{n}$ respectively, and attempt to decode the message sent by their counterpart, without error. When this is possible, that is, when there exist decoding functions $\phi_{1}:\left[2^{nR_{1}}\right]\times Y_{1}^{n}\to \left[2^{nR_{2}}\right]$ and $\phi_{2}:\left[2^{nR_{2}}\right]\times Y_{2}^{n}\to \left[2^{nR_{1}}\right]$ such that $m_{2}=\phi_{1}\left(m_{1},Y_{1}^{n}\right)$ and $m_{1}=\phi_{2}\left(m_{2},Y_{2}^{n}\right)$, for all $m_{1},m_{2}$, with probability one, then the codebook pair (or the encoding functions) is called *$\left(n,R_{1},R_{2}\right)$ uniquely decodable*. A rate pair $\left(R_{1},R_{2}\right)$ is *achievable* for the DM-TWC if an $\left(n,R_{1},R_{2}\right)$ uniquely decodable code exists for some *n*. The *non-adaptive zero-error capacity region* of a DM-TWC $P_{Y_{1},Y_{2}|X_{1},X_{2}}$ is the closure of the set of all achievable rate pairs, and is denoted here by $C_{ze}\left(P_{Y_{1},Y_{2}|X_{1},X_{2}}\right)$. Moreover, the *non-adaptive zero-error sum-capacity* of a DM-TWC $P_{Y_{1},Y_{2}|X_{1},X_{2}}$, denoted by $C_{ze}^{sum}\left(P_{Y_{1},Y_{2}|X_{1},X_{2}}\right)$, is the supremum of the sum-rate $R_{1}+R_{2}$ taken over all achievable rate pairs.

The main objective of this paper is to provide several single-letter outer and inner bounds on the non-adaptive zero-error capacity region of the DM-TWC. The remainder of this paper is organized as follows. In Section 2, we provide some necessary mathematical preliminaries, discussing in particular the characterization of zero-error DM-TWC capacity via confusion graphs, behavior under graph homomorphisms, and one-shot zero-error communication. Section 3 is devoted to three general outer bounds of the zero-error capacity region of DM-TWC, which are based on Shannon’s vanishing-error non-adaptive capacity region, a two-way analogue of the linear programming bound for point-to-point channels, and the Shannon capacity of a graph. In Section 4, we provide two general inner bounds using random coding and random linear codes respectively. In Section 5, we establish outer bounds for certain types of DM-TWC via the asymptotic spectra of graphs, and also explicitly construct the uniquely decodable codebook pairs achieving the outer bound. Some concluding remarks appear in Section 6.

## 2. Preliminaries

### 2.1. Shannon Capacity of a Graph

Let G=(V,E) be a graph with vertex set *V* and edge set *E*. Two vertices $v_{1},v_{2}$ are *adjacent*, denoted as $v_{1}∼v_{2}$, if there is an edge between $v_{1}$ and $v_{2}$, that is, $\{v_{1},v_{2}\}\in E$. An *independent set* in *G* is a subset of pairwise non-adjacent vertices. A *maximum independent set* is an independent set with the largest possible number of vertices. The size of a maximum independent set in *G* is called the *independence number* of *G*, denoted by α(G). The *complement* of a graph *G*, denoted by $\bar{G}$, is a graph with the same vertex set, where two distinct vertices of $\bar{G}$ are adjacent if and only if they are not adjacent in *G*. We write $K_{n}$ and ${\bar{K}}_{n}$ for the complete graph (containing all possible edges) and the empty graph (containing no edges) over *n* vertices, respectively.

Let G=(V(G),E(G)) and H=(V(H),E(H)) be two graphs. The *strong product* (or normal product) G⊠H of the graphs *G* and *H* is a graph such that

(1) the vertex set of G⊠H is the Cartesian product V(G)×V(H);
(2) two vertices $\left(u,u^{\prime }\right)$ and $\left(v,v^{\prime }\right)$ are adjacent if and only if one of the followings holds: (a) u=v and $u^{\prime }∼v^{\prime }$; (b) u∼v and $u^{\prime }=v^{\prime }$; (c) u∼v and $u^{\prime }∼v^{\prime }$.

The *n*-fold strong product of graph *G* with itself is denoted as $G^{n}$. The *Shannon capacity of graph G* was defined in [2] to be:

$$\Theta \left(G\right)≜\underset{n}{\sup}\frac{1}{n}\log\alpha \left(G^{n}\right)=\lim_{n\to \infty }\frac{1}{n}\log\alpha \left(G^{n}\right),$$

where the limit exists by Fekete’s lemma. We note that throughout the paper all logarithms are taken to base 2.

The *disjoint union* G⊔H of the graphs *G* and *H* is a graph such that V(G⊔H)=V(G)⊔V(H) and E(G⊔H)=E(G)⊔E(H). A *graph homomorphism* from *G* to *H*, denoted by G→H, is a mapping φ:V(G)→V(H) such that if $g_{1}∼g_{2}$ in *G*, then $\varphi \left(g_{1}\right)∼\varphi \left(g_{2}\right)$ in *H*. We write G≼H if there exists a graph homomorphism $\bar{G}\to \bar{H}$ from the complement of *G* to the complement of *H*.

In [17], Zuiddam introduced the asymptotic spectrum of graphs notion, and provided a dual characterisation of the Shannon capacity of a graph by applying Strassen’s theory of asymptotic spectra, which includes the Lovász theta number [12], the fractional clique cover number, the complement of the fractional orthogonal rank [18], and the fractional Haemers’ bound over any field [11,19,20] as specific elements of the asymptotic spectrum (also called spectral points).

**Theorem** **1** ([17])**.** *Let G be a collection of graphs that is closed under the disjoint union *⊔* and the strong product *⊠*, and also contains the graph with a single vertex $K_{1}$. Define the asymptotic spectrum Δ(G) as the set of all mappings $\eta :G\to R_{\geq 0}$ such that for all G,H∈G:*

*(1)*  *if G≼H, then η(G)≤η(H);*
*(2)*  *η(G⊔H)=η(G)+η(H);*
*(3)*  *η(G⊠H)=η(G)η(H);*
*(4)*  *$\eta (K_{1})=1$.*

*Then, $\Theta \left(G\right)=\underset{\eta \in \Delta (G)}{\inf}\log\eta \left(G\right)$. In other words, $\underset{\eta \in \Delta (G)}{\inf}\eta \left(G\right)=2^{\Theta (G)}$ and $\alpha \left(G\right)\leq \underset{\eta \in \Delta (G)}{\inf}\eta \left(G\right)$.*

As remarked in [17], $2^{\Theta (G)}$ is in general not an element of Δ(G). In fact, $2^{\Theta (G)}$ is not additive under ⊔ by a result of Alon [21], and also not multiplicative under ⊠ by a result of Haemers [11]. In Section 3.3, to derive an outer bound for zero-error capacity of a DM-TWC, we will employ the multiplicativity of η(G) for η∈Δ(G) under the ⊠ operation.

### 2.2. Confusion Graphs of Channels

In this subsection, we characterize the zero-error capacity of a discrete memoryless point-to-point channel, as well as the zero-error capacity region of a DM-TWC, in terms of suitably defined graphs. The point-to-point characterization is well known and goes back to Shannon [2], and the DM-TWC case is a natural generalization thereof.

A discrete memoryless point-to-point channel consists of a finite input alphabet X, a finite output alphabet Y, and a conditional probability distribution $P_{Y|X}\left(y|x\right)$, where x∈X, y∈Y. The channel is *memoryless* in the sense that $P_{Y_{i}|X^{i},Y^{i-1}}\left(y_{i}\right|x^{i},$ $y^{i-1})=P_{Y|X}\left(y_{i}|x_{i}\right)$ for the *i*th channel use. Suppose that a transmitter would like to convey a message $m\in [2^{nR}]$ to a receiver over the channel. To that end, the transmitter sends an input sequence $x^{n}\in X^{n}$ using an encoding function $f:\left[2^{nR}\right]\to X^{n}$, and the receiver, after observing the corresponding channel outputs $y^{n}\in Y^{n}$, guesses the message using a decoding function $\phi :Y^{n}\to \left[2^{nR}\right]$. This pair (f,ϕ) is called an *(n,R) code*, and such a code is called *uniquely decodable* if $m=\phi (y^{n})$ holds for any $m\in [2^{nR}]$ and any correspondingly possible $y^{n}$. A rate *R* is called *achievable* if an (n,R) uniquely decodable code exists for some *n*. The *zero-error capacity* of the channel is defined as the supremum of all achievable rates.

A channel $P_{Y|X}$ is associated with a *confusion graph G*, whose vertex set is the input alphabet X, and two vertices $x,x^{\prime }\in X$ are adjacent, denoted as $x∼x^{\prime }$, if and only if there exists y∈Y that is possible under both of them, that is, such that $P_{Y|X}\left(y|x\right)>0$ and $P_{Y|X}\left(y|x^{\prime }\right)>0$. It is easy to verify that C is an (n,R) uniquely decodable code if and only if C is an independent set of the graph $G^{n}$, the *n*-fold strong product of graph *G*. Consequently, the zero-error capacity of a point-to-point channel is equal to the Shannon capacity of its confusion graph *G*. Note that there are infinitely many distinct channels with the same confusion graph, and all of these channels have the same zero-error capacity.

We now proceed to similarly associate a DM-TWC with a collection of confusion graphs, which would then be shown to characterize its zero-error capacity region. To that end, note that when Alice sends a letter $x_{1}\in X_{1}$, the resulting channel from Bob back to Alice at that same instant is the point-to-point channel $P_{Y_{1}|X_{1}=x_{1},X_{2}}$. This channel is associated with a confusion graph $G_{x_{1}}$, whose vertex set is $X_{2}$ and where two vertices $x_{2},x_{2}^{\prime }\in X_{2}$ are adjacent, denoted in this case by $x_{2}\overset{x_{1}}{∼}x_{2}^{\prime }$, if and only if there exists some $y_{1}\in Y_{1}$ such that both

$$P_{Y_{1}|X_{1},X_{2}}\left(y_{1}|x_{1},x_{2}\right)>0,\;P_{Y_{1}|X_{1},X_{2}}\left(y_{1}|x_{1},x_{2}^{\prime }\right)>0,$$

where

$$P_{Y_{1}|X_{1},X_{2}}\left(y_{1}|x_{1},x_{2}\right)≜\sum_{y_{2}\in Y_{2}}P_{Y_{1},Y_{2}|X_{1},X_{2}}\left(y_{1},y_{2}|x_{1},x_{2}\right).$$

Symmetrically, when Bob sends a letter $x_{2}\in X_{2}$, the resulting channel from Alice to Bob at that same instant is associated with a confusion graph $H_{x_{2}}$, whose vertex set is $X_{1}$, and where two vertices $x_{1},x_{1}^{\prime }\in X_{1}$ are adjacent, denoted in this case by $x_{1}\overset{x_{2}}{∼}x_{1}^{\prime }$, if and only if there exists some $y_{2}\in Y_{2}$ such that both:

$$P_{Y_{2}|X_{1},X_{2}}\left(y_{2}|x_{1},x_{2}\right)>0,\;P_{Y_{2}|X_{1},X_{2}}\left(y_{2}|x_{1}^{\prime },x_{2}\right)>0,$$

where

$$P_{Y_{2}|X_{1},X_{2}}\left(y_{2}|x_{1},x_{2}\right)≜\sum_{y_{1}\in Y_{1}}P_{Y_{1},Y_{2}|X_{1},X_{2}}\left(y_{1},y_{2}|x_{1},x_{2}\right).$$

Based on the foregoing discussion, a DM-TWC $P_{Y_{1},Y_{2}|X_{1},X_{2}}$ can be decomposed into a collection of discrete memoryless point-to-point channels, and hence is associated with a corresponding collection of confusion graphs, denoted by $[G_{1},\ldots ,G_{|X_{1}|};H_{1},$ $\ldots ,H_{|X_{2}|}]$, where $V\left(G_{1}\right)=\cdots =V\left(G_{|X_{1}|}\right)=X_{2}$ and $V\left(H_{1}\right)=\cdots =V\left(H_{|X_{2}|}\right)=X_{1}$. The following useful observation is immediate, and in particular shows that the zero-error capacity region of a DM-TWC is a function of its confusion graphs only. Thus, from here and on, we will sometimes identify the channel with its collection of confusion graphs.

**Proposition** **1.** *Consider a DM-TWC $P_{Y_{1},Y_{2}|X_{1},X_{2}}$, associated with the collection of confusion graphs $[G_{1},\ldots ,G_{|X_{1}|};$ $H_{1},\ldots ,$ $H_{|X_{2}|}]$. A codebook pair (A,B) is uniquely decodable for $P_{Y_{1},Y_{2}|X_{1},X_{2}}$ if and only if for any $a^{n}=\left(a_{1},\ldots ,a_{n}\right)\in A$ and $b^{n}=\left(b_{1},\ldots ,b_{n}\right)\in B$, it holds that B is an independent set of $G_{a_{1}}⊠\cdots ⊠G_{a_{n}}$, and A is an independent set of $H_{b_{1}}⊠\cdots ⊠H_{b_{n}}$.*

In particular, we see that the capacity region $C_{ze}\left(P_{Y_{1},Y_{2}|X_{1},X_{2}}\right)$ depends only on the corresponding confusion graphs $[G_{1},\ldots ,G_{|X_{1}|};H_{1},$ $\ldots ,H_{|X_{2}|}]$. Hence, in the sequel, we will write $C_{ze}\left(\left[G_{1},\ldots ,G_{|X_{1}|};H_{1},\ldots ,H_{|X_{2}|}\right]\right)$ and $C_{ze}^{sum}([G_{1},\ldots ,G_{|X_{1}|};H_{1},$ $\ldots ,H_{|X_{2}|}])$ to represent $C_{ze}\left(P_{Y_{1},Y_{2}|X_{1},X_{2}}\right)$ and $C_{ze}^{sum}\left(P_{Y_{1},Y_{2}|X_{1},X_{2}}\right)$, respectively. We will also often identify the channel with its confusion graphs, and refer to it as $\left[\left\{G_{i}\right\};\left\{H_{j}\right\}\right]$, when it is clear from the context. This also leads to the following immediate observation, analogues to the point-to-point case.

**Proposition** **2.** *If $P_{Y_{1},Y_{2}|X_{1},X_{2}}$ and $Q_{Y_{1},Y_{2}|X_{1},X_{2}}$ have the same confusion graphs up to some relabeling on input symbols, then $C_{ze}\left(P_{Y_{1},Y_{2}|X_{1},X_{2}}\right)=C_{ze}\left(Q_{Y_{1},Y_{2}|X_{1},X_{2}}\right)$.*

This further immediately implies:

**Proposition** **3.** *$C_{ze}\left(P_{Y_{1},Y_{2}|X_{1},X_{2}}\right)$ depends only on the conditional marginal distributions $P_{Y_{1}|X_{1},X_{2}}$ and $P_{Y_{2}|X_{1},X_{2}}$.*

The strong product of two DM-TWCs $\left[G_{1},\ldots ,G_{|X_{1}|};H_{1},\ldots ,H_{|X_{2}|}\right]$ and $[G_{1}^{\prime },\ldots ,$ $G_{|X_{1}^{\prime }|}^{\prime };$ $H_{1}^{\prime },\ldots ,H_{|X_{2}^{\prime }|}^{\prime }]$, denoted by $\left[\left\{G_{i}\right\};\left\{H_{j}\right\}\right]⊠\left[\left\{G_{i}^{\prime }\right\};\left\{H_{j}^{\prime }\right\}\right]$, refers to a DM-TWC having input alphabets $X_{1}\times X_{1}^{\prime }$ and $X_{2}\times X_{2}^{\prime }$, as well as confusion graphs

$$[\left\{G_{i}⊠G_{i^{\prime }}^{\prime }:\;i\in X_{1},i^{\prime }\in X_{1}^{\prime }\right\};\left\{H_{j}⊠H_{j^{\prime }}^{\prime }:\;j\in X_{2},j^{\prime }\in X_{2}^{\prime }\right\}].$$

Considering the zero-error sum-capacity with respect to the strong product, we have the lemma below.

**Lemma** **1.**
$$C_{ze}^{sum}\left(\left[\left\{G_{i}\right\};\left\{H_{j}\right\}\right]⊠\left[\left\{G_{i}^{\prime }\right\};\left\{H_{j}^{\prime }\right\}\right]\right)\geq C_{ze}^{sum}\left([\left\{G_{i}\right\};\left\{H_{j}\right\}]\right)+C_{ze}^{sum}\left([\left\{G_{i}^{\prime }\right\};\left\{H_{j}^{\prime }\right\}]\right).$$

**Proof.**  To prove this lemma, it is sufficient to prove that, for any $\left(n,R_{1},R_{2}\right)$ (resp. $(n,R_{1}^{\prime },$ $R_{2}^{\prime })$) uniquely decodable codebook pair (A,B) (resp. $\left(A^{\prime },B^{\prime }\right)$) for channel $\left[\left\{G_{i}\right\};\left\{H_{j}\right\}\right]$ (resp. $\left[\left\{G_{i}^{\prime }\right\};\left\{H_{j}^{\prime }\right\}\right]$), there exists an $\left(n,R_{1}+R_{1}^{\prime },R_{2}+R_{2}^{\prime }\right)$ uniquely decodable codebook pair for the associated product channel $\left[\left\{G_{i}\right\};\left\{H_{j}\right\}\right]⊠\left[\left\{G_{i}^{\prime }\right\};\left\{H_{j}^{\prime }\right\}\right]$. To that end, let

$$\begin{array}{cc} A^{*} & =\{\left(\left(a_{1},a_{1}^{\prime }\right),\ldots ,\left(a_{n},a_{n}^{\prime }\right)\right):a^{n}\in A,a^{\prime n}\in A^{\prime }\},\\ B^{*} & =\{\left(\left(b_{1},b_{1}^{\prime }\right),\ldots ,\left(b_{n},b_{n}^{\prime }\right)\right):b^{n}\in B,b^{\prime n}\in B^{\prime }\}. \end{array}$$

It is easy to verify that $\left(A^{*},B^{*}\right)$ is uniquely decodable for the product channel. Moreover, $|A^{*}\left|=|A|\right|A^{\prime }|=2^{n(R_{1}+R_{1}^{\prime })}$ and $|B^{*}\left|=|B|\right|B^{\prime }|=2^{n(R_{2}+R_{2}^{\prime })}$. The lemma follows. □

### 2.3. Dual Graph Homomorphisms

In this subsection we study the behavior of the zero-error capacity region of a DM-TWC under graph homomorphisms, generalizing a similar analysis from the point-to-point channel case [2]. Let $\left[\left\{G_{i}\right\};\left\{H_{j}\right\}\right]$ and $\left[\left\{G_{i}^{\prime }\right\};\left\{H_{j}^{\prime }\right\}\right]$ be two collections of confusion graphs corresponding to two DM-TWCs such that $V\left(G_{i}\right)=V\left(G\right)$, $V\left(H_{j}\right)=V\left(H\right)$, $V\left(G_{i}^{\prime }\right)=V\left(G^{\prime }\right)$ and $V\left(H_{j}^{\prime }\right)=V\left(H^{\prime }\right)$. A *dual graph homomorphism* from $\left[\left\{G_{i}\right\};\left\{H_{j}\right\}\right]$ to $\left[\left\{G_{i}^{\prime }\right\};\left\{H_{j}^{\prime }\right\}\right]$, denoted by $\left[\left\{G_{i}\right\};\left\{H_{j}\right\}\right]\to \left[\left\{G_{i}^{\prime }\right\};\left\{H_{j}^{\prime }\right\}\right]$, is a pair of mappings (φ,ψ), where $\varphi :V\left(H\right)\to V\left(H^{\prime }\right)$ and $\psi :V\left(G\right)\to V\left(G^{\prime }\right)$, such that

(1) if $v_{1}∼v_{2}$ in $G_{i}$, then $\psi \left(v_{1}\right)∼\psi \left(v_{2}\right)$ in $G_{\varphi (i)}^{\prime }$; and
(2) if $u_{1}∼u_{2}$ in $H_{j}$, then $\varphi \left(u_{1}\right)∼\varphi \left(u_{2}\right)$ in $H_{\psi (j)}^{\prime }$.

It is easy to see that the dual graph homomorphism is a natural generalization of the standard graph homomorphism of two graphs, in the sense that they are both adjacency preserving. We write $\left[\left\{G_{i}\right\};\left\{H_{j}\right\}\right]⪯\left[\left\{G_{i}^{\prime }\right\};\left\{H_{j}^{\prime }\right\}\right]$ if there exists a dual graph homomorphism from $\left[\left\{{\bar{G}}_{i}\right\};\left\{{\bar{H}}_{j}\right\}\right]$ to $\left[\left\{{\bar{G}}_{i}^{\prime }\right\};\left\{{\bar{H}}_{j}^{\prime }\right\}\right]$. Then:

**Lemma** **2.** *If $\left[\left\{G_{i}\right\};\left\{H_{j}\right\}\right]⪯\left[\left\{G_{i}^{\prime }\right\};\left\{H_{j}^{\prime }\right\}\right]$, and ${\bar{G}}_{i}$ and ${\bar{H}}_{j}$ do not have self-loops, then*

$$C_{ze}\left(\left[\left\{G_{i}\right\};\left\{H_{j}\right\}\right]\right)\subseteq C_{ze}\left(\left[\left\{G_{i}^{\prime }\right\};\left\{H_{j}^{\prime }\right\}\right]\right).$$

**Proof.**  Suppose $\left(\varphi ,\psi \right):\left[\left\{{\bar{G}}_{i}\right\};\left\{{\bar{H}}_{j}\right\}\right]\to \left[\left\{{\bar{G}}_{i}^{\prime }\right\};\left\{{\bar{H}}_{j}^{\prime }\right\}\right]$ and (A,B) is a uniquely decodable codebook pair of length *n* for the DM-TWC $\left[\left\{G_{i}\right\};\left\{H_{j}\right\}\right]$. Let

$$\begin{array}{cc} \Phi (A) & =\{\varphi \left(a^{n}\right)=\left(\varphi \left(a_{1}\right),\ldots ,\varphi \left(a_{n}\right)\right):a^{n}\in A\},\\ \Psi (B) & =\{\psi \left(b^{n}\right)=\left(\psi \left(b_{1}\right),\ldots ,\psi \left(b_{n}\right)\right):b^{n}\in B\}. \end{array}$$

We now show that (Φ(A),Ψ(B)) is a uniquely decodable codebook pair for the DM-TWC $\left[\left\{G_{i}^{\prime }\right\};\left\{H_{j}^{\prime }\right\}\right]$. To that end, it suffices to show that for any distinct $a^{n},{\tilde{a}}^{n}\in A$ and $b^{n},{\tilde{b}}^{n}\in B$, we have

(1) $$\begin{array}{cc} \varphi (a^{n}) & ≁\varphi \left({\tilde{a}}^{n}\right)\;\mathrm{in}\;H_{\psi (b_{1})}⊠\cdots ⊠H_{\psi (b_{n})},\\ \psi (b^{n}) & ≁\psi \left({\tilde{b}}^{n}\right)\;\mathrm{in}\;G_{\varphi (a_{1})}⊠\cdots ⊠G_{\varphi (a_{n})}. \end{array}$$

Indeed, since (A,B) is a uniquely decodable codebook pair, there exist coordinates i,j∈[n] such that $a_{i}≁{\tilde{a}}_{i}$ in $H_{b_{i}}$ and $b_{j}≁{\tilde{b}}_{j}$ in $G_{a_{j}}$. By the definition of (φ,ψ), we have that $\varphi \left(a_{i}\right)≁\varphi \left({\tilde{a}}_{i}\right)$ in $H_{\psi (b_{i})}$ and $\psi \left(b_{j}\right)≁\psi \left({\tilde{b}}_{j}\right)$ in $G_{\varphi (a_{j})}$, implying (1). It is also evident that |Φ(A)|=|A| and |Ψ(B)|=|B|. The lemma now follows by taking the union over all uniquely decodable codebook pairs (A,B) for $\left[\left\{G_{i}\right\};\left\{H_{j}\right\}\right]$. □

### 2.4. One-Shot Zero-Error Communication

In this subsection, we consider the problem of zero-error communication over a DM-TWC with only a single channel use by the two parties (i.e., n=1). We refer to the associated set of achievable rate pairs as the *one-shot zero-error capacity region*, and the associated sum-rate as the *one-shot zero-error sum-capacity*. Recall that the one-shot zero-error capacity of a point-to-point channel is simply the logarithm of the independence number of its confusion graph; this quantity yields a lower bound on the zero-error capacity of the channel, and also provides an infinite-letter expression for the capacity when evaluated over the product graph. It is therefore interesting to study the analogue of the independence number in the two-way case, which in particular would yield an inner bound on the zero-error capacity region of the DM-TWC. For simplicity of exposition, we will focus here on the one-shot zero-error sum-capacity only.

For convenience we define some notions first. Let $\left[\left\{G_{i}\right\};\left\{H_{j}\right\}\right]$ be a DM-TWC such that $V\left(G_{i}\right)=X_{2}$ and $V\left(H_{j}\right)=X_{1}$. A pair (S,T) of subsets $S\subseteq X_{1}$ and $T\subseteq X_{2}$ is called a *dual clique pair* of the DM-TWC if $t\overset{s}{∼}t^{\prime }$ and $s\overset{t}{∼}s^{\prime }$ for any distinct $s,s^{\prime }\in S$ and distinct $t,t^{\prime }\in T$, that is, *S* is a clique in each $H_{t}$ for t∈T, and *T* is a clique in each $G_{s}$ for s∈S. A pair (S,T) of subsets $S\subseteq X_{1}$ and $T\subseteq X_{2}$ is called a *dual independent pair* of the DM-TWC if *T* is an independent set of the graph $G_{s}$ for each s∈S, and *S* is an independent set of the graph $H_{t}$ for each t∈T. A *maximum dual independent pair* is a dual independent pair (S,T) with the largest possible product of sizes |S||T|. This product is called the *independence product* of $\left[\left\{G_{i}\right\};\left\{H_{j}\right\}\right]$, denoted by $\pi (\left\{G_{i}\right\};\left\{H_{j}\right\})$. According to the definition, the one-shot zero-error sum-capacity of the DM-TWC is $\log\pi (\left\{G_{i}\right\};\left\{H_{j}\right\})$. It is also readily seen that if two channels have the same confusion graphs up to some relabeling of input symbols, then they have the same collections of dual clique pairs and dual independent pairs, and hence the same one-shot zero-error sum-capacity.

For two graphs $G_{1}$ and $G_{2}$, let $G_{1}\cup G_{2}$ be the *union* of $G_{1}$ and $G_{2}$ such that $V\left(G_{1}\cup G_{2}\right)=V\left(G_{1}\right)\cup V\left(G_{2}\right)$ and $E\left(G_{1}\cup G_{2}\right)=E\left(G_{1}\right)\cup E\left(G_{2}\right)$. Notice that the graph disjoint union ⊔ in Section 2.1 is a special case of the union ∪, when the vertex sets of $G_{1}$ and $G_{2}$ are disjoint. For notation convenience, in the rest of this subsection we let $|X_{1}|=m_{1}$ and $|X_{2}|=m_{2}$. The following simple observations are now in order.

**Proposition** **4.** *Suppose (S,T) is a dual independent pair of $\left[G_{1},\ldots ,G_{m_{1}};H_{1},\ldots ,H_{m_{2}}\right]$. Then:*

*(1)*  *If |S|=1, then $\left|T\right|\leq \max_{1\leq i\leq m_{1}}\alpha \left(G_{i}\right)$. The equality holds by taking S={s} and T be a maximum independent set of $G_{s}$, where $s\in \arg\max_{1\leq i\leq m_{1}}\alpha \left(G_{i}\right)$.*
*(2)*  *$\left|S\right|\leq \min_{t\in T}\alpha \left(H_{t}\right)$.*
*(3)*  *S is an independent set of $\;{⋃}_{t\in T}H_{t}$.*

**Proof.**  The results follow directly from the definition of dual independent pairs. □

**Lemma** **3.** *Let $\left[G_{1},\ldots ,G_{m_{1}};H_{1},\ldots ,H_{m_{2}}\right]$ be a DM-TWC and G, H be graphs such that $V\left(G\right)=X_{2}$, $V\left(H\right)=X_{1}$. Then:*

*(1)* 
$\max\left\{\max_{1\leq i\leq m_{1}}\alpha \left(G_{i}\right),\max_{1\leq j\leq m_{2}}\alpha \left(H_{j}\right)\right\}\leq \pi \left(G_{1},\ldots ,G_{m_{1}};H_{1},\ldots ,H_{m_{2}}\right)\leq \max_{1\leq i\leq m_{1}}\alpha \left(G_{i}\right)\cdot \max_{1\leq j\leq m_{2}}\alpha \left(H_{j}\right).$

*(2)* 
π(G,…,G;H,…,H)=α(G)α(H).

*(3)* 
$\pi \left({\bar{K}}_{m_{2}},G,\ldots ,G;{\bar{K}}_{m_{1}},H,\ldots ,H\right)=\max\left\{\alpha \left(G\right)\alpha \left(H\right),m_{1},m_{2}\right\}.$

*(4)*  *$\pi \left(G_{1},\ldots ,G_{m_{1}};K_{m_{1}},\ldots ,K_{m_{1}}\right)=\max_{1\leq i\leq m_{1}}\alpha \left(G_{i}\right)$.*

**Proof.**  (1) The lower bound follows from Proposition 4 (1) and the symmetry of *S* and *T*. From Proposition 4 (2), we have

$$\begin{array}{cc} \left|S\right| & \leq \min_{t\in T}\alpha \left(H_{t}\right)\leq \max_{1\leq j\leq m_{2}}\alpha \left(H_{j}\right),\\ \left|T\right| & \leq \min_{s\in S}\alpha \left(G_{s}\right)\leq \max_{1\leq i\leq m_{1}}\alpha \left(G_{i}\right), \end{array}$$

yielding the upper bound.

(2) From claim (1) above, we have π(G,…,G;H,…,H)≤α(G)α(H). The equality holds by taking *S* and *T* as the maximum independent sets of *H* and *G* respectively.

(3) From claims (1) and (2) above, we have

$$\pi \left({\bar{K}}_{m_{2}},G,\ldots ,G;{\bar{K}}_{m_{1}},H,\ldots ,H\right)\geq \max\left\{\alpha \left(G\right)\alpha \left(H\right),m_{1},m_{2}\right\}.$$

On the other hand, suppose (S,T) is a dual independent pair. We have the following three cases: (i) If |S|=1 then by Proposition 4, claim (1), we have $\left|S||T\right|\leq m_{2}$. (ii) If |T|=1, similar to case (i), we have $\left|S||T\right|\leq m_{1}$. (iii) If |S|≥2 and |T|≥2, then by Proposition 4, claim (2), we obtain |S||T|≤α(G)α(H). Thus $\pi \left({\bar{K}}_{m_{2}},G,\ldots ,G;{\bar{K}}_{m_{1}},H,\ldots ,H\right)\leq \max\left\{\alpha \left(G\right)\alpha \left(H\right),m_{1},m_{2}\right\}$.

(4) is a direct consequence of claim (1) above. The lemma follows. □

By graph homomorphisms we immediately have:

**Proposition** **5.** *If $\left[\left\{G_{i}\right\};\left\{H_{j}\right\}\right]⪯\left[\left\{G_{i}^{\prime }\right\};\left\{H_{j}^{\prime }\right\}\right]$, then*

$$\pi \left(\left\{G_{i}\right\};\left\{H_{j}\right\}\right)\leq \pi \left(\left\{G_{i}^{\prime }\right\};\left\{H_{j}^{\prime }\right\}\right).$$

Next, we shall provide an upper bound for $\pi (\left\{G_{i}\right\};\left\{H_{j}\right\})$ via a generalization of the Lovász theta number [12]. Let Γ be an arbitrary $\left(m_{1}+m_{2}\right)\times \left(m_{1}+m_{2}\right)$ positive semi-definite matrix (i.e., Γ⪰0), and $\Gamma_{i,j}$ be its (i,j)th entry. Let *J* be an $m_{1}\times m_{2}$ all-one matrix, and $I_{n}$ be an n×n identity matrix. For any matrices *A* and *B*, denote 〈A,B〉=trace(AB) and denote $A^{T}$ as the transpose of matrix *A*. Now define $\rho (\left\{G_{i}\right\},\left\{H_{j}\right\})$ as

(2) $$\begin{array}{cc} \mathrm{maximize}\; & \langle \left(\begin{array}{cc} 0 & J\\ J^{T} & 0 \end{array}\right),\Gamma \rangle \\ \mathrm{subject}\;\mathrm{to}\; & \langle \left(\begin{array}{cc} I_{m_{1}} & 0\\ 0 & 0 \end{array}\right),\Gamma \rangle =1,\\ \; & \langle \left(\begin{array}{cc} 0 & 0\\ 0 & I_{m_{2}} \end{array}\right),\Gamma \rangle =1,\\ \; & \Gamma_{i,j+m_{1}}\cdot \Gamma_{i,k+m_{1}}=0,\;\;\;\forall \;i\in X_{1},\;j,k\in X_{2},\;j\neq k,\;j∼k\;\mathrm{in}\;G_{i}\\ \; & \Gamma_{i+m_{1},j}\cdot \Gamma_{i+m_{1},k}=0,\;\;\;\forall \;i\in X_{2},\;j,k\in X_{1},\;j\neq k,\;j∼k\;\mathrm{in}\;H_{i}\\ \; & \Gamma_{i,k+m_{1}}\cdot \Gamma_{j,k+m_{1}}=0,\;\;\;\forall \;i,j\in X_{1},\;k\in X_{2},\;i\neq j,\;i∼j\;\mathrm{in}\;H_{k}\\ \; & \Gamma_{i+m_{1},k}\cdot \Gamma_{j+m_{1},k}=0,\;\;\;\forall \;i,j\in X_{2},\;k\in X_{1},\;i\neq j,\;i∼j\;\mathrm{in}\;G_{k}\\ \; & \Gamma ⪰0. \end{array}$$

**Lemma** **4.** *$\pi \left(\left\{G_{i}\right\},\left\{H_{j}\right\}\right)\leq {\left(\frac{1}{2}\rho \left(\left\{G_{i}\right\},\left\{H_{j}\right\}\right)\right)}^{2}$.*

**Proof.**  Suppose (S,T) with $S\subseteq X_{1}$, $T\subseteq X_{2}$ is a maximum dual independent pair such that $\left|S||T|=\pi (\left\{G_{i}\right\},\left\{H_{j}\right\}\right)$. For a number *m* and a set *S*, denote m+S={m+s:s∈S}. Let Γ be an $\left(m_{1}+m_{2}\right)\times \left(m_{1}+m_{2}\right)$ matrix such that

$$\Gamma_{i,j}=\left\{\begin{array}{cc} \frac{1}{\left|S\right|}, & \mathrm{if}\;i\in S,\;j\in S\\ \frac{1}{\sqrt{\left|S||T\right|}}, & \mathrm{if}\;i\in S,\;j\in m_{1}+T,\;\mathrm{or}\;\;i\in m_{1}+T,\;j\in S\\ \frac{1}{\left|T\right|}, & \mathrm{if}\;i\in m_{1}+T,\;j\in m_{1}+T\\ 0, & \mathrm{otherwise}. \end{array}\right.$$

Notice that for any vector $x^{m_{1}+m_{2}}=\left(x_{1},\ldots ,x_{m_{1}+m_{2}}\right)$ we have

$$x^{m_{1}+m_{2}}\cdot \Gamma \cdot {\left(x^{m_{1}+m_{2}}\right)}^{T}={\left(\frac{1}{\sqrt{\left|S\right|}}\sum_{i\in S}x_{i}+\frac{1}{\sqrt{\left|T\right|}}\sum_{j\in T}x_{m_{1}+j}\right)}^{2}\geq 0.$$

This shows that Γ is a positive semi-definite matrix satisfying the equality constraints in (2). Accordingly, Γ is a feasible solution for program (2) and

$$\begin{array}{cc} \rho (\left\{G_{i}\right\},\left\{H_{j}\right\}) & \geq \langle \left(\begin{array}{cc} 0 & J\\ J^{T} & 0 \end{array}\right),\Gamma \rangle \\ \; & =2\sqrt{\left|S||T\right|}\\ \; & =2\sqrt{\pi (\left\{G_{i}\right\},\left\{H_{j}\right\})}, \end{array}$$

implying the result. This completes the proof. □

### 2.5. Information-Theoretic Notations

We recall some standard information-theoretic quantities that will be used in the sequel. Let X,Y be two discrete random variables taking values from sets X,Y according to a joint probability distribution $P_{XY}$. Let $P_{X}$ denote the marginal probability distribution for *X*, where $P_{X}\left(x\right)=\sum_{y\in Y}P_{XY}\left(x,y\right)$, and $P_{Y}$ be the marginal probability distribution for *Y* similarly. The Shannon *entropy* of *X* is denoted by H(X) where $H\left(X\right)=-\sum_{x\in X}P_{X}\left(x\right)\log P_{X}\left(x\right)$. In particular, the *binary entropy function* is written as h(x)=−xlogx−(1−x)log(1−x), where 0≤x≤1. The *conditional entropy* of *X* given *Y* is written as H(X|Y) where $H\left(X|Y\right)=-P_{XY}\left(x,y\right)\log\frac{P_{XY}\left(x,y\right)}{P_{Y}\left(y\right)}$. The *mutual information* between *X* and *Y* is I(X;Y)=H(X)−H(X|Y). The *conditional mutual information* of X,Y given another random variable *Z* is I(X;Y|Z)=H(X|Z)−H(X|Y,Z). The following basic properties will be used in the arguments afterwards.

**Proposition** **6.** *(1) H(X)≥0,I(X;Y)≥0. (Non-negativity)*

*(2) H(X|Y)≤H(X), I(X;Y|Z)≤I(X;Y). (Conditioning reduces entropy)*

*(3) $H\left(X_{1},X_{2},\ldots ,X_{n}\right)=\sum_{i=1}^{n}H\left(X_{i}|X_{1},\ldots ,X_{i-1}\right)$. (Entropy chain rule)*

## 3. Outer Bounds

In this section, we provide single-letter outer bounds for the non-adaptive zero-error capacity region of the DM-TWC. First in Section 3.1, we present two simple outer bounds, one based on Shannon’s vanishing-error non-adaptive capacity region and the other on a two-way analogue of the linear programming bound for point-to-point channels. Next in Section 3.2, we combine the two bounds given in Section 3.1 and obtain an outer bound that is generally better than both. Finally, in Section 3.3 we derive another single-letter outer bound via the asymptotic spectra of graphs.

### 3.1. Simple Bounds

It is trivial to see that Shannon’s vanishing-error non-adaptive capacity region of the DM-TWC ([1], Theorem 3) contains its zero-error counterpart. First recall Shannon’s bound in [1].

**Lemma** **5** ([1])**.** *The vanishing-error non-adaptive capacity region of a DM-TWC $P_{Y_{1},Y_{2}|X_{1},X_{2}}$ is the convex hull of the set:*

$$\underset{P_{X_{1}},P_{X_{2}}}{⋃}\left\{\left(R_{1},R_{2}\right):R_{1}\geq 0,R_{2}\geq 0,R_{1}=I\left(X_{1};Y_{2}|X_{2}\right),R_{2}=I\left(X_{2};Y_{1}|X_{1}\right)\right\}$$

*where the union is taken over all product input probability distributions $P_{X_{1}}\times P_{X_{2}}$.*

Together with Proposition 2, this immediately yields the following outer bound.

**Lemma** **6.** *$C_{ze}\left(P_{Y_{1},Y_{2}|X_{1},X_{2}}\right)$ is contained in*

(3) $$\begin{array}{cc} \underset{Q_{Y_{1},Y_{2}|X_{1},X_{2}}}{⋂}\underset{0\leq \lambda \leq 1}{⋂}\{(R_{1}, & R_{2}):R_{1}\geq 0,R_{2}\geq 0,\lambda R_{1}+\left(1-\lambda \right)R_{2}\leq \max_{P_{X_{1}},P_{X_{2}}}\epsilon \left(\lambda \right)\}, \end{array}$$

*where*

(4) $$\epsilon \left(\lambda \right)≜\lambda I\left(X_{1};Y_{2}|X_{2}\right)+\left(1-\lambda \right)I\left(X_{2};Y_{1}|X_{1}\right).$$

*The first intersection is taken over all DM-TWCs $Q_{Y_{1},Y_{2}|X_{1},X_{2}}$ with the same adjacency as $P_{Y_{1},Y_{2}|X_{1},X_{2}}$, and the maximum is taken over all product input probability distributions $P_{X_{1}}\times P_{X_{2}}$.*

**Remark** **1.** The bound (3) can also be written in the standard form

$$\begin{array}{cc} \underset{Q_{Y_{1},Y_{2}|X_{1},X_{2}}}{⋂}\underset{P_{X_{1}},P_{X_{2}}}{⋃}\{\left(R_{1},R_{2}\right):\; & R_{1}\geq 0,R_{2}\geq 0,\\ \; & R_{1}\leq I\left(X_{1};Y_{2}|X_{2}\right),\\ \; & R_{2}\leq I\left(X_{2};Y_{1}|X_{1}\right)\}. \end{array}$$

Here we prefer however to use the form (3), for ease of comparison with forthcoming bounds.

We now proceed to obtain a combinatorial outer bound. Recall that a dual clique pair of a DM-TWC is a pair (S,T) of subsets $S\subseteq X_{1}$ and $T\subseteq X_{2}$ such that $t\overset{s}{∼}t^{\prime }$ and $s\overset{t}{∼}s^{\prime }$ for any distinct $s,s^{\prime }\in S$ and distinct $t,t^{\prime }\in T$. In the sequel, we adopt the convention that $0^{0}=1$.

**Lemma** **7.** *$C_{ze}\left(P_{Y_{1},Y_{2}|X_{1},X_{2}}\right)$ is contained in:*

(5) $$\underset{0\leq \lambda \leq 1}{⋂}\left\{\left(R_{1},R_{2}\right):R_{1}\geq 0,R_{2}\geq 0,\lambda R_{1}+\left(1-\lambda \right)R_{2}\leq \max_{P_{X_{1}},P_{X_{2}}}-\log l\left(\lambda \right)\right\},$$

*where*

(6) $$l\left(\lambda \right)≜\max_{S,T}{\left(\sum_{x_{1}\in S}P_{X_{1}}\left(x_{1}\right)\right)}^{\lambda }{\left(\sum_{x_{2}\in T}P_{X_{2}}\left(x_{2}\right)\right)}^{1-\lambda }$$

*and the maximum in *(5)* is taken over all the input probability distributions $P_{X_{1}}$ and $P_{X_{2}}$, and the maximum in *(6)* is taken over all the dual clique pairs (S,T) of $P_{Y_{1},Y_{2}|X_{1},X_{2}}$.*

**Proof.**  Let (A,B) be a uniquely decodable codebook pair of length *n*. We will show that:

(7) $${\left|A\right|}^{\lambda }{\left|B\right|}^{1-\lambda }\leq \kappa \cdot {\left(\frac{1}{l(\lambda )}\right)}^{n}$$

by induction on *n*, where κ is a constant independent of *n*.

Indeed, for the base case n=1, one could take subsets $A\subseteq X_{1}$, $B\subseteq X_{2}$ such that for any distinct $a,a^{\prime }\in A$ and distinct $b,b^{\prime }\in B$, we have $a\overset{b}{≁}a^{\prime }$ and $b\overset{a}{≁}b^{\prime }$. Clearly, $\left|A||B|\leq \right|X_{1}\left|\right|X_{2}|$ and (7) follows by taking κ sufficiently large.

Assume that (7) holds for every length $n^{\prime }\leq n-1$, and let us proceed to prove for length *n*. Suppose $\left(A,B\right)\subseteq X_{1}^{n}\times X_{2}^{n}$ is a uniquely decodable codebook pair of length *n*. For a vector $x^{n}$, let $x^{n\setminus i}≜\left(x_{1},\ldots ,x_{i-1},x_{i+1},\ldots ,x_{n}\right)$ be its projection over all coordinates not equal to *i*. For each coordinate 1≤i≤n and each $x_{1}\in X_{1}$, $x_{2}\in X_{2}$, let

(8) $$\begin{array}{cc} A_{i}\left(x_{1}\right) & ≜\{a^{n\setminus i}:\;a^{n}\in A,\;a_{i}=x_{1}\},\\ B_{i}\left(x_{2}\right) & ≜\{b^{n\setminus i}:\;b^{n}\in B,\;b_{i}=x_{2}\} \end{array}$$

be the projections of each codebook obtained by fixing the *i*th coordinate. Define the distributions induced by these projections over $X_{1}$ and $X_{2}$ respectively to be

(9) $$P_{X_{1}}^{i}\left(x_{1}\right)≜\frac{|A_{i}\left(x_{1}\right)|}{\left|A\right|},\;P_{X_{2}}^{i}\left(x_{2}\right)≜\frac{|B_{i}\left(x_{2}\right)|}{\left|B\right|}.$$

Furthermore, for any two subsets $S\subseteq X_{1}$ and $T\subseteq X_{2}$, define the codebooks induced by the unions over *S* and *T* of the respective projected codebooks, to be

(10) $$A_{i}\left(S\right)≜\underset{x_{1}\in S}{⋃}A_{i}\left(x_{1}\right),\;B_{i}\left(T\right)≜\underset{x_{2}\in T}{⋃}B_{i}\left(x_{2}\right).$$

Note that if (S,T) is a dual clique pair such that $A_{i}\left(S\right)\neq \emptyset$ and $B_{i}\left(T\right)\neq \emptyset$, then the unions in (10) are disjoint, as otherwise this would contradict the assumption that (A,B) is uniquely decodable. Hence

(11) $$\begin{array}{c} |A_{i}\left(S\right)|=\sum_{x_{1}\in S}|A_{i}\left(x_{1}\right)\left|,\;\right|B_{i}\left(T\right)|=\sum_{x_{2}\in T}\left|B_{i}\left(x_{2}\right)\right|, \end{array}$$

and also, for any i∈[n] it must hold that $\left(A_{i}\left(S\right),B_{i}\left(T\right)\right)$ is a uniquely decodable codebook pair of length n−1. Combining (8), (9) and (11) gives

(12) $$\begin{array}{cc} {\left|A\right|}^{\lambda }{\left|B\right|}^{1-\lambda } & ={\left(\frac{|A_{i}\left(S\right)|}{\sum_{s\in S}P_{X_{1}}^{i}\left(s\right)}\right)}^{\lambda }\cdot {\left(\frac{|B_{i}\left(T\right)|}{\sum_{t\in T}P_{X_{2}}^{i}\left(t\right)})\right)}^{1-\lambda }. \end{array}$$

By the inductive hypothesis, we obtain

$$\begin{array}{cc} {\left|A\right|}^{\lambda }{\left|B\right|}^{1-\lambda }\; & \leq \frac{\kappa \cdot {\left(\frac{1}{l(\lambda )}\right)}^{n-1}}{{\left(\sum_{s\in S}P_{X_{1}}^{i}\left(s\right)\right)}^{\lambda }\cdot {\left(\sum_{t\in T}P_{X_{2}}^{i}\left(t\right)\right)}^{1-\lambda }}\\ \; & \leq \frac{\kappa \cdot {\left(\frac{1}{l(\lambda )}\right)}^{n-1}}{l(\lambda )}=\kappa \cdot {\left(\frac{1}{l(\lambda )}\right)}^{n}, \end{array}$$

where the second inequality follows from the definition of l(λ) in (6). This completes the proof. □

The following is a trivial corollary of Lemmas 6 and 7.

**Corollary** **1.** *$C_{ze}\left(P_{Y_{1},Y_{2}|X_{1},X_{2}}\right)$ is contained in*

(13) $$\begin{array}{cc} \underset{Q_{Y_{1},Y_{2}|X_{1},X_{2}}}{⋂}\underset{0\leq \lambda \leq 1}{⋂}\{(R_{1}, & R_{2}):R_{1}\geq 0,R_{2}\geq 0,\lambda R_{1}+\left(1-\lambda \right)R_{2}\leq t\left(\lambda \right)\}, \end{array}$$

*where*

(14) $$t\left(\lambda \right)≜\min\left\{\max_{P_{X_{1}},P_{X_{2}}}\epsilon \left(\lambda \right),\max_{P_{X_{1}},P_{X_{2}}}-\log l\left(\lambda \right)\right\}.$$

### 3.2. An Improved Bound

We now provide a single-letter outer bound, in which the order of the minimum and the maximum in (14) is swapped. This generally yields a tighter outer bound due to the max–min inequality. In fact, our bound can be seen as a generalization of the one obtained by Holzman and Körner for the binary multiplying channel [13], in which case the max–min is indeed strictly tighter than the min–max.

**Theorem** **2.** *$C_{ze}\left(P_{Y_{1},Y_{2}|X_{1},X_{2}}\right)$ is contained in*

(15) $$\begin{array}{cc} \underset{Q_{Y_{1},Y_{2}|X_{1},X_{2}}}{⋂}\underset{0\leq \lambda \leq 1}{⋂}\{(R_{1}, & R_{2}):R_{1}\geq 0,R_{2}\geq 0,\lambda R_{1}+\left(1-\lambda \right)R_{2}\leq \theta \left(\lambda \right)\}, \end{array}$$

*where*

(16) $$\theta \left(\lambda \right)≜\max_{P_{X_{1}},P_{X_{2}}}\min\left\{\epsilon \left(\lambda \right),-\log l\left(\lambda \right)\right\}.$$

*The first intersection is taken over all DM-TWCs $Q_{Y_{1},Y_{2}|X_{1},X_{2}}$ with the same adjacency as $P_{Y_{1},Y_{2}|X_{1},X_{2}}$, and the maximum is taken over all product input probability distributions $P_{X_{1}}\times P_{X_{2}}$.*

**Proof.**  The intersection over all $Q_{Y_{1},Y_{2}|X_{1},X_{2}}$ follows from Proposition 2. Hence without loss of generality, we prove that for $P_{Y_{1},Y_{2}|X_{1},X_{2}}$, each achievable rate pair $\left(R_{1},R_{2}\right)$ satisfies $\lambda R_{1}+\left(1-\lambda \right)R_{2}\leq \theta \left(\lambda \right)$, where 0≤λ≤1.

To that end, for each uniquely decodable codebook pair (A,B) of length *n*, we will show that:

(17) $${\left|A\right|}^{\lambda }{\left|B\right|}^{1-\lambda }\leq \kappa \cdot 2^{n\theta (\lambda )}$$

by induction on *n*, where κ is a constant independent of *n*. The base case of n=1, follows in the same way as in the proof of the base case in Lemma 7. Assume that (17) holds for all length $n^{\prime }\leq n-1$, and let us prove it also holds for length *n*. Suppose that $\left(A,B\right)\subseteq X_{1}^{n}\times X_{2}^{n}$ is a uniquely decodable codebook pair of length *n*. Following the same steps of (8)–(12) in the argument of Lemma 7, we also have:

(18) $$\begin{array}{cc} {\left|A\right|}^{\lambda }{\left|B\right|}^{1-\lambda } & ={\left(\frac{|A_{i}\left(S\right)|}{\sum_{s\in S}P_{X_{1}}^{i}\left(s\right)}\right)}^{\lambda }\cdot {\left(\frac{|B_{i}\left(T\right)|}{\sum_{t\in T}P_{X_{2}}^{i}\left(t\right)})\right)}^{1-\lambda }. \end{array}$$

Now, if there exists a dual clique pair (S,T) and a coordinate 1≤i≤n such that

(19) $${\left(\sum_{s\in S}P_{X_{1}}^{i}\left(s\right)\right)}^{\lambda }{\left(\sum_{t\in T}P_{X_{2}}^{i}\left(t\right)\right)}^{1-\lambda }\geq 2^{-\theta (\lambda )},$$

then (18) implies

$$\begin{array}{c} {\left|A\right|}^{\lambda }{\left|B\right|}^{1-\lambda }\leq \frac{\kappa 2^{\left(n-1)\theta (\lambda \right)}}{2^{-\theta (\lambda )}}=\kappa 2^{n\theta (\lambda )}, \end{array}$$

where the inequality follows from the inductive hypothesis and (19). Therefore, we conclude that (17) holds under condition (19).

Assume now that condition (19) is not satisfied, that is,

(20) $$\max_{i\in [n]}\max_{S,T}{\left(\sum_{s\in S}P_{X_{1}}^{i}\left(s\right)\right)}^{\lambda }{\left(\sum_{t\in T}P_{X_{2}}^{i}\left(t\right)\right)}^{1-\lambda }<2^{-\theta (\lambda )}.$$

Let $A^{n}$ and $B^{n}$ be codewords chosen from A and B respectively, uniformly at random, and let $Y_{1}^{n}$, $Y_{2}^{n}$ be the corresponding channel outputs. Since (A,B) is a uniquely decodable codebook pair of length *n*, it must be that:

(21) $$\begin{array}{cc} \log|A| & =I(Y_{2}^{n};A^{n}|B^{n}),\\ \log|B| & =I(Y_{1}^{n};B^{n}|A^{n}). \end{array}$$

On the other hand, we have:

(22) $$\begin{array}{cc} I(Y_{1}^{n};B^{n}|A^{n}) & =H\left(Y_{1}^{n}|A^{n}\right)-H\left(Y_{1}^{n}|A^{n},B^{n}\right) \end{array}$$

(23) $$\begin{array}{cc} & =\sum_{i=1}^{n}H\left(Y_{1,i}|Y_{1,1},\ldots ,Y_{1,i-1},A^{n}\right)-\sum_{i=1}^{n}H\left(Y_{1,i}|A_{i},B_{i}\right) \end{array}$$

(24) $$\begin{array}{cc} & \leq \sum_{i=1}^{n}H\left(Y_{1,i}|A_{i}\right)-\sum_{i=1}^{n}H\left(Y_{1,i}|A_{i},B_{i}\right) \end{array}$$

(25) $$\begin{array}{cc} \; & =\sum_{i=1}^{n}I\left(Y_{1,i};B_{i}|A_{i}\right), \end{array}$$

where (23) follows from the entropy chain rule and the memorylessness of the channel, and (24) follows from the fact that conditioning reduces entropy. Similarly,

(26) $$I\left(Y_{2}^{n};A^{n}|B^{n}\right)\leq \sum_{i=1}^{n}I\left(Y_{2,i};A_{i}|B_{i}\right).$$

Combining (20)–(26), we obtain

(27) log|A|λ|B|1−λ=λlog|A|+(1−λ)log|B|≤∑i=1nλI(Y2,i;Ai|Bi)+(1−λ)I(Y1,i;Bi|Ai)≤maxPX1,PX2,l(λ)<2−θ(λ)n[λI(Y2;X1|X2)+(1−λ)I(Y1;X2|X1)]=maxPX1,PX2,l(λ)<2−θ(λ)n·ϵ(λ),

where ϵ(λ) and l(λ) are defined in (4) and (6), respectively, and the maximum is taken over all product input probability distributions $P_{X_{1}}\times P_{X_{2}}$ such that $l\left(\lambda \right)<2^{-\theta (\lambda )}$, following condition (20).

By the definition of θ(λ), we have:

(28) $$\begin{array}{cc} \theta (\lambda ) & =\max_{P_{X_{1}},P_{X_{2}}}\min\left\{\epsilon \left(\lambda \right),-\log l\left(\lambda \right)\right\} \end{array}$$

(29) ≥maxPX1,PX2,l(λ)<2−θ(λ)min{ϵ(λ),−logl(λ)}.

Note that for any input distributions $P_{X_{1}},P_{X_{2}}$ such that $l\left(\lambda \right)<2^{-\theta (\lambda )}$, we have

(30) −logl(λ)>θ(λ).

Combining (29) and (30), we obtain

(31) maxPX1,PX2,l(λ)<2−θ(λ)ϵ(λ)≤θ(λ).

Substituting (31) into (27), we have ${\log|A|}^{\lambda }{\left|B\right|}^{1-\lambda }\leq n\theta \left(\lambda \right)$, completing the proof. □

We remark that Theorem 2 immediately implies, in particular, the following upper bound on the zero-error capacity of the point-to-point discrete memoryless channel.

**Corollary** **2.** *The zero-error capacity of the discrete memoryless channel $P_{Y|X}$ is upper bounded by*

$$\min_{Q_{Y|X}}\;\max_{P_{X}}\;\min\left\{I\left(X;Y\right),-\log\max_{C}\sum_{x\in C}P_{X}\left(x\right)\right\}.$$

*The outer minimum is taken over all the $Q_{Y|X}$ having the same confusion graph as $P_{Y|X}$, the outer maximum is taken over all the input distributions $P_{X}$, and the inner maximum is taken over all the cliques C of the confusion graph of the channel.*

As it turns out, the upper bound in Corollary 2 coincides with the linear programming bound on the zero-error capacity of a point-to-point discrete memoryless channel in [2]. Namely,

$$\min_{Q_{Y|X}}\;\max_{P_{X}}I\left(X;Y\right)=\max_{P_{X}}\left\{-\log\max_{C}\sum_{x\in C}P_{X}\left(x\right)\right\}$$

for any point-to-point discrete memoryless channel $P_{Y|X}$. This fact was originally conjectured by Shannon [2] and later proved by Ahlswede [22]. In other words, this means that in the point-to-point case, Corollary 1 yields exactly the same bound as Theorem 2. However, this is not the case in general for the DM-TWC. For example, recall that Holzman and Körner [13] derived the bound in Theorem 2 in the special case of the (deterministic) binary multiplying channel (using λ=0.5) and numerically showed that it is strictly better than what can be obtained from Corollary 1. Next we give another example showing that Theorem 2 outperforms Corollary 1 for a noisy (i.e., non-deterministic) DM-TWC as well.

**Example** **1.** Let $X_{1}=\left\{0,1,2\right\},X_{2}=Y_{1}=Y_{2}=\left\{0,1\right\}$, and the conditional probability distribution $P_{Y_{1},Y_{2}|X_{1},X_{2}}$ be
$y_{1}y_{2}$$x_{1}x_{2}$ 00  01 10 11  20  21 00110000010000101000δ00111001−δ100
where δ∈(0,1). Corollary 1 gives the upper bound

$$R_{1}+R_{2}\leq \min\left\{\max_{P_{X_{1}},P_{X_{2}}}\epsilon^{*},\max_{P_{X_{1}},P_{X_{2}}}-\log l^{*}\right\}\approx 1.2933,$$

where

$$\begin{array}{cc} \epsilon^{*} & =I\left(X_{1};Y_{2}|X_{2}\right)+I\left(X_{2};Y_{1}|X_{1}\right)\\ \; & =P_{X_{1}}\left(2\right)\cdot h\left(P_{X_{2}}\left(0\right)\right)+P_{X_{2}}\left(0\right)\cdot h\left(P_{X_{1}}\left(0\right)+\delta \cdot P_{X_{1}}\left(1\right)\right)-P_{X_{1}}\left(1\right)\cdot P_{X_{2}}\left(0\right)\cdot h\left(\delta \right)\\ \; & \;\;\;+P_{X_{2}}\left(1\right)\cdot h\left(P_{X_{1}}\left(1\right)\right),\\ l^{*} & =\max_{S,T}\left(\sum_{x_{1}\in S}P_{X_{1}}\left(x_{1}\right)\right)\left(\sum_{x_{2}\in T}P_{X_{2}}\left(x_{2}\right)\right)\\ \; & =\max\{P_{X_{1}}\left(0\right),P_{X_{1}}\left(1\right),P_{X_{2}}\left(0\right)\cdot \left(P_{X_{1}}\left(0\right)+P_{X_{1}}\left(1\right)\right),P_{X_{2}}\left(0\right)\cdot \left(P_{X_{1}}\left(1\right)+P_{X_{1}}\left(2\right)\right),\\ \; & \;\;P_{X_{2}}\left(1\right)\cdot \left(P_{X_{1}}\left(0\right)+P_{X_{1}}\left(2\right)\right)\}, \end{array}$$

and h(x)=−xlogx−(1−x)log(1−x). In contrast, Theorem 2 yields a tighter upper bound of

$$R_{1}+R_{2}\leq \max_{P_{X_{1}},P_{X_{2}}}\min\left\{\epsilon^{*},-\log l^{*}\right\}\approx 1.2910.$$

### 3.3. An Outer Bound via Shannon Capacity of a Graph

Based on Lemma 3 and the Shannon capacity of a graph, we immediately have the following bound.

**Lemma** **8.**
$$\begin{array}{c} C_{ze}^{sum}\left(\left[G_{1},\ldots ,G_{|X_{1}|};H_{1},\ldots ,H_{|X_{2}|}\right]\right)\leq \max_{x_{1}\in X_{1},x_{2}\in X_{2}}\Theta \left(G_{x_{1}}\right)+\Theta \left(H_{x_{2}}\right). \end{array}$$

It is worth noting that the above bound could be optimal in the sense that when all $G_{i}=G$ and $H_{j}=H$, it is easily verified that $C_{ze}^{sum}\left(\left[G,\ldots ,G;H,\ldots ,H\right]\right)=\Theta \left(G\right)+\Theta \left(H\right)$. However, the bound in Lemma 8 is not tight in general. Later in Section 5, we will improve the bound in Lemma 8 for certain scenarios and show that the improved bound (in Theorem 5) could outperform Theorem 2 (see Example 3), and be achieved in special cases (see Theorem 7).

## 4. Inner Bounds

In this section, we present two inner bounds for the non-adaptive zero-error capacity region of the DM-TWC, one based on random coding and the other on linear codes.

### 4.1. Random Coding

The random coding for DM-TWC is standard and generalizes a known bound by Shannon for the one-way case [2]. To obtain the random coding inner bound, we need the following lemma from [1].

**Lemma** **9** ([1])**.** *Let X be a random variable taking values in [N], and ${\left\{f_{i}:\left[N\right]\to R_{+}\right\}}_{i\in [d]}$ be a collection of nonnegative functions. Then there exists x∈[N] such that $f_{i}\left(x\right)\leq d\cdot E\left[f_{i}\left(X\right)\right]$ for all i∈[d].*

**Theorem** **3.** *$C_{ze}\left(P_{Y_{1},Y_{2}|X_{1},X_{2}}\right)$ contains the region:*

(32) ⋃PX1,PX2{(R1,R2):R1≥0,R2≥0,R1≤−12log∑x1∼x2x1′∨x1=x1′,x1,x1′∈X1,x2∈X2PX1(x1)PX1(x1′)PX2(x2),R2≤−12log∑x2∼x1x2′∨x2=x2′,x1∈X1,x2,x2′∈X2PX1(x1)PX2(x2)PX2(x2′),}

*where the union is taken over all input distributions $P_{X_{1}}$, $P_{X_{2}}$.*

**Proof.**  We randomly draw a codebook pair (A,B), such that A (resp. B) consists of $M_{1}$ (resp.$\;M_{2}$) statistically independent words, where each word is generated i.i.d. according to a probability distribution $P_{X_{1}}$ (resp.$\;P_{X_{2}}$). A word $a^{n}\in A$ is called *bad*, if there exist two words, $b^{n},{\tilde{b}}^{n}\in B$ that are either equal or adjacent in $G_{a_{1}}⊠\cdots ⊠G_{a_{n}}$. For any particular words $a^{n}\in A$, $b^{n},{\tilde{b}}^{n}\in B$ and coordinate i∈[n], the probability that $b_{i}∼{\tilde{b}}_{i}$ in $G_{a_{i}}$ is upper bounded by:

∑x2∼x1x2′∨x2=x2′,x1∈X1,x2,x2′∈X2PX1(x1)PX2(x2)PX2(x2′).

Since all the coordinates are independent, the probability that $b^{n}∼{\tilde{b}}^{n}$ in $G_{a_{1}}⊠\cdots ⊠G_{a_{n}}$ is at most:

(33) ∑x2∼x1x2′∨x2=x2′,x1∈X1,x2,x2′∈X2PX1(x1)PX2(x2)PX2(x2′)n.

Denote by $\mathrm{Bad}(a^{n})$ the number of 2-subsets $\{b^{n},{\tilde{b}}^{n}\}\subseteq B$ such that $b^{n}∼{\tilde{b}}^{n}$ in $G_{a_{1}}⊠\cdots ⊠G_{a_{n}}$. Then,

Pr{anisbad}=Pr{Bad(an)≥1}≤E[Bad(an)]≤M22∑x2∼x1x2′∨x2=x2′,x1∈X1,x2,x2′∈X2PX1(x1)PX2(x2)PX2(x2′)n,

where the first inequality is by Markov’s inequality, and the second inequality follows from (33) and the linearity of expectation. Similarly, a word $b^{n}\in B$ is called *bad*, if there exist two words $a^{n},{\tilde{a}}^{n}\in A$ that are equal or adjacent in $H_{b_{1}}⊠\cdots ⊠H_{b_{n}}$, and we have

Pr{bnisbad}≤M12∑x1∼x2x1′∨x1=x1′,x1,x1′∈X1,x2∈X2PX1(x1)PX1(x1′)PX2(x2)n.

Let $f_{1}\left(A,B\right)$, $f_{2}\left(A,B\right)$ be the number of bad words in A and B respectively. Then, we have:

(34) E[f1(A,B)]≤M1M22∑x2∼x1x2′∨x2=x2′,x1∈X1,x2,x2′∈X2PX1(x1)PX2(x2)PX2(x2′)n,

(35) E[f2(A,B)]≤M2M12∑x1∼x2x1′∨x1=x1′,x1,x1′∈X1,x2∈X2PX1(x1)PX1(x1′)PX2(x2)n.

By Lemma 9, there exists a pair $\left(A^{*},B^{*}\right)$ such that

(36) $$f_{1}\left(A^{*},B^{*}\right)\leq 2E\left[f_{1}\left(A,B\right)\right],\;f_{2}\left(A^{*},B^{*}\right)\leq 2E\left[f_{2}\left(A,B\right)\right].$$

Remove all the bad words in $A^{*}$ and $B^{*}$ respectively, yielding a codebook pair $\left(A^{\prime },B^{\prime }\right)$ such that:

(37) $$|A^{\prime }|=M_{1}-f_{1}\left(A^{*},B^{*}\right)\;\;\;\mathrm{and}\;\;\;\left|B^{\prime }\right|=M_{2}-f_{2}\left(A^{*},B^{*}\right).$$

It is readily seen that $\left(A^{\prime },B^{\prime }\right)$ is a uniquely decodable codebook pair.

Now let

(38) M1=(1−ξ1)n2∑x1∼x2x1′∨x1=x1′,x1,x1′∈X1,x2∈X2PX1(x1)PX1(x1′)PX2(x2)−n2,

(39) M2=(1−ξ2)n2∑x2∼x1x2′∨x2=x2′,x1∈X1,x2,x2′∈X2PX1(x1)PX2(x2)PX2(x2′)−n2,

where $\xi_{1},\xi_{2}$ are arbitrarily small positive numbers. Combining (34)–(39), we obtain:

|A′|≥(1−(1−ξ2)n)(1−ξ1)n2∑x1∼x2x1′∨x1=x1′,x1,x1′∈X1,x2∈X2PX1(x1)PX1(x1′)PX2(x2)−n2,|B′|≥(1−(1−ξ1)n)(1−ξ2)n2∑x2∼x1x2′∨x2=x2′,x1∈X1,x2,x2′∈X2PX1(x1)PX2(x2)PX2(x2′)−n2.

Since $\xi_{1},\xi_{2}$ are arbitrarily small, by taking *n* as sufficiently large, we can find an $\left(n,R_{1},R_{2}\right)$ uniquely decodable codebook pair arbitrarily close to (32), as desired. □

### 4.2. Linear Codes

In this subsection, we present a construction of uniquely decodable codes via linear codes, which generalizes a known result for the binary multiplying channel [15]. Let us introduce some notations first. Suppose *D* is a set of letters, $x^{n}$ and $y^{n}$ are vectors of length *n*, and C is a collection of vectors of length *n*. Let:

(40) $${\mathrm{ind}}_{D}\left(x^{n}\right)≜\left\{1\leq i\leq n:x_{i}\in D\right\}$$

denote the collection of indices where $x_{i}\in D$. For I⊆[n] let $y^{n}{|}_{I}$ denote the vector obtained from $y^{n}$ by projecting onto the coordinates in *I*, and denote

$$C_{|I}≜\left\{c^{n}{|}_{I}:c^{n}\in C\right\}.$$

Let $P_{Y_{1},Y_{2}|X_{1},X_{2}}$ be a DM-TWC. We say that $x_{1}\in X_{1}$ is a *detecting symbol*, if $x_{2}\overset{x_{1}}{≁}x_{2}^{\prime }$ for any distinct $x_{2},x_{2}^{\prime }\in X_{2}$. A detecting symbol $x_{2}\in X_{2}$ is defined analogously. Let $D_{1}\subseteq X_{1}$ and $D_{2}\subseteq X_{2}$ denote the sets of all detecting symbols in $X_{1}$ and $X_{2}$, respectively. A vector $a^{n}\in X_{1}^{n}$ is called a *detecting vector* for $B\subseteq X_{2}^{n}$ if

(41) $$\left|B_{|{\mathrm{ind}}_{D_{1}}\left(a^{n}\right)}\right|=\left|B\right|.$$

Similarly, a vector $b^{n}\in X_{2}^{n}$ is a *detecting vector* for $A\subseteq X_{1}^{n}$ if

(42) $$|A_{|{\mathrm{ind}}_{D_{2}}\left(b^{n}\right)}|=|A|.$$

The following claim is immediate.

**Proposition** **7.** *Let $A\subseteq X_{1}^{n}$, $B\subseteq X_{2}^{n}$. If each $a^{n}\in A$ is a detecting vector for B and each $b^{n}\in B$ is a detecting vector for A, then (A,B) is a uniquely decodable codebook pair.*

Proposition 7 provides a sufficient condition for a uniquely decodable code, which is not always necessary (see Example 2). Nevertheless, this sufficient condition furnishes us with a way of constructing uniquely decodable codes by employing linear codes.

**Example** **2.** Suppose that $X_{1}=\left\{a_{0},a_{1},a_{2}\right\}$, $X_{2}=\left\{b_{0},b_{1}\right\}$ such that $D_{1}=\left\{a_{0},a_{1},a_{2}\right\}$, $D_{2}=\left\{b_{1}\right\}$, and $a_{0}\overset{b_{0}}{∼}a_{1}$, $a_{0}\overset{b_{0}}{∼}a_{2}$, $a_{1}\overset{b_{0}}{≁}a_{2}$. Let $A=\{a_{0}a_{0}a_{0},a_{1}a_{1}a_{1},a_{0}a_{1}a_{2}\}$ and $B=\{b_{0}b_{1}b_{0}\}$. It is easy to verify that (A,B) is a uniquely decodable codebook pair. However, ${\mathrm{ind}}_{D_{2}}\left(b_{0}b_{1}b_{0}\right)=\left\{2\right\}$ and $|A_{\left|\{2\right\}}\left|=\right|\left\{a_{0},a_{1}\right\}|=2<|A|=3$, implying that $b_{0}b_{1}b_{0}$ is not a detecting vector for A.

Assume that $|X_{1}|=q_{1}$ and $|X_{2}|=q_{2}$, where $q_{1},q_{2}$ are prime powers, and let us think of the alphabets as $F_{q_{1}}$ and $F_{q_{2}}$, respectively. The following theorem gives an inner bound on the capacity region, which is a generalization of the Tolhuizen’s construction for the Blackwell’s multiplying channel [15].

**Theorem** **4.** *Let $P_{Y_{1},Y_{2}|X_{1},X_{2}}$ be a DM-TWC with input alphabet sizes $|X_{1}|=q_{1}$, $|X_{2}|=q_{2}$, where $q_{1}$, $q_{2}$ are prime powers. If $X_{1}$ and $X_{2}$ contain $\tau_{1}$ and $\tau_{2}$ detecting symbols respectively, then $C_{ze}\left(P_{Y_{1},Y_{2}|X_{1},X_{2}}\right)$ contains the region*

(43) $$\begin{array}{cc} \underset{0\leq \alpha ,\beta \leq 1}{⋃}\{\left(R_{1},R_{2}\right):\; & R_{1}\geq 0,R_{2}\geq 0,\\ \; & R_{1}\leq h\left(\alpha \right)+\alpha \log\tau_{2}+\left(1-\alpha \right)\log\left(q_{2}-\tau_{2}\right)-\left(1-\beta \right)\log q_{2},\\ \; & R_{2}\leq h\left(\beta \right)+\beta \log\tau_{1}+\left(1-\beta \right)\log\left(q_{1}-\tau_{1}\right)-\left(1-\alpha \right)\log q_{1}\}, \end{array}$$

*where h(x)≜−xlogx−(1−x)log(1−x) is the binary entropy function.*

To prove this theorem, we need the following lemma. The case that $q_{1}=q_{2}=2$ and τ=1 was proved in ([15], Theorem 3). Lemma 10 follows from similar argument.

**Lemma** **10.** *Let q, $q^{\prime }$ be prime powers, n,k be positive integers such that 1≤k≤n, and $D\subseteq F_{q^{\prime }}$ with cardinality |D|=τ. Then there exists a pair (C,Y(C)) satisfying that:*

*(1)*  *C is a q-ary [n,k] linear code;*
*(2)*  *$Y\left(C\right)\subseteq F_{q^{\prime }}^{n}$ such that*
|Y(C)|≥nkτk(q′−τ)n−k∏i=1∞(1−q−i);
*(3)*  *for each $x^{n}\in Y\left(C\right)$, we have $|{ind}_{D}\left(x^{n}\right)|=k$ and $\left|C_{|{ind}_{D}\left(x^{n}\right)}\right|=\left|C\right|$.*

**Proof.**  Let *A* be a k×n matrix of full rank over $F_{q}$, then $C\left(A\right)≜\left\{y^{k}A:y^{k}\in F_{q}^{k}\right\}$ is a *q*-ary [n,k] linear code generated by *A*. Recall that for every $x^{n}\in F_{q^{\prime }}^{n}$, ${\mathrm{ind}}_{D}\left(x^{n}\right)=\left\{i\in \left[n\right]:\;x_{i}\in D\right\}$ as in (40). Denote:

$$Y\left(C\left(A\right)\right)≜\left\{x^{n}\in F_{q^{\prime }}^{n}:\;|{\mathrm{ind}}_{D}\left(x^{n}\right)\left|=k,\;\right|C_{|{\mathrm{ind}}_{D}\left(x^{n}\right)}\left|=|C\right|\right\}.$$

Let ${A|}_{{\mathrm{ind}}_{D}\left(x^{n}\right)}$ denote the $k\times |{\mathrm{ind}}_{D}\left(x^{n}\right)|$ submatrix of *A* with columns indexed by ${\mathrm{ind}}_{D}\left(x^{n}\right)$. It is easy to see that $|C_{|{\mathrm{ind}}_{D}\left(x^{n}\right)}\left|=|C\right|$ is equivalent to $\mathrm{rank}(A{|}_{{\mathrm{ind}}_{D}\left(x^{n}\right)})=k$. Denote:

(44) $$P≜\left\{\left(A,x^{n}\right):\;A\in F_{q}^{k\times n},\;x^{n}\in F_{q^{\prime }}^{n},\;\left|{\mathrm{ind}}_{D}\left(x^{n}\right)\right|=k,\;\mathrm{rank}\left(A{|}_{{\mathrm{ind}}_{D}\left(x^{n}\right)}\right)=k\right\},$$

and let us proceed by double counting the cardinality of P.

On the one hand, the number of vectors $x^{n}\in F_{q^{\prime }}^{n}$ such that $|{\mathrm{ind}}_{D}\left(x^{n}\right)|=k$ is nkτk(q′−τ)n−k. For each such $x^{n}$, there are $q^{k(n-k)}I_{q}\left(k\right)$ corresponding k×n matrices $A\in F_{q}^{k\times n}$ such that $\mathrm{rank}(A{|}_{{\mathrm{ind}}_{D}\left(x^{n}\right)})=k$, where $I_{q}\left(k\right)=\prod_{i=0}^{k-1}\left(q^{k}-q^{i}\right)$ is the number of k×k invertible matrices over $F_{q}$, see ([15], Lemma 3). Hence, we have:

|P|=nkτk(q′−τ)n−kqk(n−k)Iq(k).

On the other hand, the number of matrices $A\in F_{q}^{k\times n}$ is $q^{nk}$. By (44) and the pigeonhole principle, there exist a matrix $A^{*}\in F_{q}^{k\times n}$ and a corresponding code $C(A^{*})$ such that $\left|Y\left(C\left(A\right)\right)\right|\geq \left|P\right|/q^{nk}$. Letting $C=C(A^{*})$, the lemma follows. □

**Proof** **of Theorem 4.** For i=1,2, let us identify $X_{i}$ with $F_{q_{i}}$, and let the respective sets of all detecting symbols be $D_{i}\subseteq F_{q_{i}}$ with $|D_{i}|=\tau_{i}$.

To prove the existence of a uniquely decodable codebook pair based on Proposition 7, we first use Lemma 10 to find two “one-sided” uniquely decodable linear codebook pairs, and then combine them to the desired codebook pair by employing their cosets in $F_{q_{1}}^{n}$ and $F_{q_{2}}^{n}$.

First, letting $q=q_{1}$, $q^{\prime }=q_{2}$, $G=D_{2}$ and $\tau =\tau_{2}$ in Lemma 10, we have a pair $\left(C_{1},Y\left(C_{1}\right)\right)$ satisfying that $C_{1}$ is a $q_{1}$-ary $\left[n,k_{1}\right]$ linear code and $Y\left(C_{1}\right)\subseteq F_{q_{2}}^{n}$ such that

(45) |Y(C1)|≥nk1τ2k1(q2−τ2)n−k1∏i=1∞(1−q1−i).

Similarly, letting $q=q_{2}$, $q^{\prime }=q_{1}$, $G=D_{1}$ and $\tau =\tau_{1}$ in Lemma 10, we have a pair $\left(C_{2},Y\left(C_{2}\right)\right)$ satisfying that $C_{2}$ is a $q_{2}$-ary $\left[n,k_{2}\right]$ linear code and $Y\left(C_{2}\right)\subseteq F_{q_{1}}^{n}$ such that

(46) |Y(C2)|≥nk2τ1k2(q1−τ1)n−k2∏i=1∞(1−q2−i).

The property (3) in Lemma 10 implies that each $x^{n}\in Y\left(C_{i}\right)$ is a detecting vector for $C_{i}$ for i=1,2. Note that if $\Xi \left(C_{i}\right)\subseteq F_{q_{i}}^{n}$ is a coset of $C_{i}$, then each $x^{n}\in Y\left(C_{i}\right)$ is also a detecting vector for $\Xi (C_{i})$.

Now we are going to combine the two pairs $\left(C_{1},Y\left(C_{1}\right)\right)$ and $\left(C_{2},Y\left(C_{2}\right)\right)$. Since $C_{i}$ has $q_{i}^{n-k_{i}}$ cosets, then by the pigeonhole principle there exists coset $\Xi (C_{i})$ of $C_{i}$ such that:

(47) $$\begin{array}{cc} A & ≜Y\left(C_{1}\right)\cap \Xi \left(C_{2}\right),\;\left|A\right|\geq \frac{\left|Y(C_{1})\right|}{q_{2}^{n-k_{2}}},\\ B & ≜Y\left(C_{2}\right)\cap \Xi \left(C_{1}\right),\;\left|B\right|\geq \frac{\left|Y(C_{2})\right|}{q_{1}^{n-k_{1}}}. \end{array}$$

We now notice that each vector in A (resp.B) is a detecting vector for B (resp.A), hence by Proposition 7 (A,B) is a uniquely decodable codebook pair. Moreover, for fixed $q_{1}$, $q_{2}$, we have:

$$\begin{array}{cc} \frac{\log|A|}{n}\geq & \;h\left(\frac{k_{1}}{n}\right)+\left(\frac{k_{1}}{n}\right)\log\tau_{2}+\left(1-\frac{k_{1}}{n}\right)\log\left(q_{2}-\tau_{2}\right)-\left(1-\frac{k_{2}}{n}\right)\log q_{2}\;-\;O\left(\frac{1}{n}\right), \end{array}$$

$$\begin{array}{cc} \frac{\log|B|}{n}\geq & \;h\left(\frac{k_{2}}{n}\right)+\left(\frac{k_{2}}{n}\right)\log\tau_{1}+\left(1-\frac{k_{2}}{n}\right)\log\left(q_{1}-\tau_{1}\right)-\left(1-\frac{k_{1}}{n}\right)\log q_{1}\;-\;O\left(\frac{1}{n}\right), \end{array}$$

which follows from (45)–(47). Letting $\alpha =k_{1}/n\in \left[0,1\right]$, $\beta =k_{2}/n\in \left[0,1\right]$, we obtain:

$$\begin{array}{cc} R_{1} & =\lim_{n\to \infty }\frac{\log|A|}{n}\geq h\left(\alpha \right)+\alpha \log\tau_{2}+\left(1-\alpha \right)\log\left(q_{2}-\tau_{2}\right)-\left(1-\beta \right)\log q_{2},\\ R_{2} & =\lim_{n\to \infty }\frac{\log|B|}{n}\geq h\left(\beta \right)+\beta \log\tau_{1}+\left(1-\beta \right)\log\left(q_{1}-\tau_{1}\right)-\left(1-\alpha \right)\log q_{1}. \end{array}$$

Therefore, (43) follows, as desired. □

We note that for any DM-TWC, one could only exploit part of input symbols $X_{1}^{\prime }\subseteq X_{1}$, $X_{2}^{\prime }\subseteq X_{2}$ to meet the requirements in Theorem 4. Hence we in fact have the following more general bound.

**Corollary** **3.** *Let $P_{Y_{1},Y_{2}|X_{1},X_{2}}$ be a DM-TWC with input alphabets $X_{1}$, $X_{2}$. Then $C_{ze}\left(P_{Y_{1},Y_{2}|X_{1},X_{2}}\right)$ contains the region:*

⋃X1′⊆X1,X2′⊆X2⋃0≤α,β≤1{(R1,R2):R1≥0,R2≥0,R1≤h(α)+αlogτ2′+(1−α)log(q2′−τ2′)−(1−β)logq2′,R2≤h(β)+βlogτ1′+(1−β)log(q1′−τ1′)−(1−α)logq1′},

*where the first union is taken over all $X_{1}^{\prime }\subseteq X_{1}$, $X_{2}^{\prime }\subseteq X_{2}$ such that $|X_{1}^{\prime }|$ and $|X_{2}^{\prime }|$ are prime powers, and contain $\tau_{1}^{\prime }$ and $\tau_{2}^{\prime }$ detecting symbols, respectively.*

Notice that the region (43) relies on the number $q_{1}$, $q_{2}$ of symbols being used and the corresponding numbers $\tau_{1},\tau_{2}$ of detecting symbols. It is thus possible that using only a smaller subset of channel inputs would yield higher achievable rates (when using our linear coding strategy) than those obtained by using larger subsets. For Example 1, Corollary 3 shows that a lower bound on the maximum sum-rate $R_{1}+R_{2}$ is 1.17, which is better than the random coding lower bound 1.0907.

## 5. Certain Types of DM-TWC

In this section, we consider the DM-TWC in the scenario that the communication in one direction is stable (in particular, noiseless). First we briefly review the probabilistic refinement of the Shannon capacity of a graph in Section 5.1. Then in Section 5.2, we provide an outer bound on the zero-error capacity region via the asymptotic spectrum of graphs. In Section 5.3, we present explicit constructions that attain our outer bound in certain special cases.

### 5.1. Probabilistic Refinement of the Shannon Capacity of a Graph

We first recall some basic notions and results from the method-of-types. Let $x^{n}\in X^{n}$ be a sequence and $N(a|x^{n})$ be the number of times that a∈X appears in sequence $x^{n}$. The *type* $P_{x^{n}}$ of $x^{n}$ is the relative proportion of each symbol in X, that is, $P_{x^{n}}\left(a\right)≜\frac{N(a|x^{n})}{n}$ for all a∈X. Let $P_{n}$ denote the collection of all possible types of sequences of length *n*. For every $P\in P_{n}$, the *type class*$T^{n}\left(P\right)$ of *P* is the set of sequences of type *P*, that is, $T^{n}\left(P\right)≜\left\{x^{n}:P_{x^{n}}=P\right\}$. The *ϵ-typical set* of *P* is

$$T_{\epsilon }^{n}\left(P\right)≜\{x^{n}\in X^{n}:|P_{x^{n}}\left(a\right)-P\left(a\right)\left|<\epsilon ,\;\forall \;a\in X\right\}.$$

The *joint type* $P_{x^{n},y^{n}}$ of a pair of sequences $\left(x^{n},y^{n}\right)$ is the relative proportion of occurrences of each pair of symbols of X×Y, that is, $P_{x^{n},y^{n}}≜\frac{N(a,b|x^{n},y^{n})}{n}$ for all a∈X and b∈Y. By the Bayes’ rule, the *conditional type* $P_{x^{n}|y^{n}}$ is defined as:

$$P_{x^{n}|y^{n}}\left(a,b\right)≜\frac{N(a,b|x^{n},y^{n})}{N(b|y^{n})}=\frac{P_{x^{n},y^{n}}\left(a,b\right)}{P_{y^{n}}\left(b\right)}.$$

**Lemma** **11** ([23])**.** *$|P_{n}{|\leq \left(n+1\right)}^{\left|X\right|}$.*

**Lemma** **12** ([23])**.** *$\forall \;P\in P_{n}$, we have $\frac{2^{nH(X)}}{{\left(n+1\right)}^{\left|X\right|}}\leq \left|T^{n}\left(P\right)\right|\leq 2^{nH(X)}$.*

In [24], Csiszár and Körner introduced the probabilistic refinement of the Shannon capacity of a graph, imposing that the independent set consists of sequences of the (asymptotically) same type. Let $G_{\epsilon }^{n}\left[P\right]$ denote the subgraph of $G^{n}$ induced by $T_{\epsilon }^{n}\left(P\right)$. The Shannon capacity of graph *G* relative to *P* is defined as

$$\Theta \left(G,P\right)≜\lim_{\epsilon \to 0}\underset{n\to \infty }{lim sup}\frac{1}{n}\log\alpha \left(G_{\epsilon }^{n}\left[P\right]\right).$$

Let $G^{n}\left[P\right]$ denote the subgraph of $G^{n}$ induced by $T^{n}\left(P\right)$. Then, it is readily seen that:

$$\underset{n\to \infty }{lim sup}\frac{1}{n}\log\alpha \left(G^{n}\left[P\right]\right)\leq \Theta \left(G,P\right).$$

For each η∈Δ(G), define

(48) $$\hat{\eta }\left(G,P\right)≜\lim_{\epsilon \to 0}\underset{n\to \infty }{lim sup}\frac{1}{n}\log\eta \left(G_{\epsilon }^{n}\left[P\right]\right).$$

If $G={\bar{K}}_{n}$, then according to Lemma 12, we have

(49) $$\hat{\eta }\left({\bar{K}}_{n},P\right)=H\left(X\right)$$

for any η∈Δ(G). Very recently, Vrana [25] proved the following results on $\hat{\eta }\left(G,P\right)$.

**Lemma** **13** ([25])**.** *The limit in (48) exists and*

*(1)*  *$\Theta \left(G,P\right)=\min_{\eta \in \Delta (G)}\hat{\eta }\left(G,P\right)$;*
*(2)*  *$\log\eta \left(G\right)=\max_{P}\;\hat{\eta }\left(G,P\right)$ for η∈Δ(G).*

According to Lemma 11, it is easily seen that:

$$\Theta \left(G\right)=\max_{P}\;\Theta \left(G,P\right).$$

Here, we would like to mention that the probabilistic refinement of the Lovász theta number was introduced and investigated by Marton in [26] via a non-asymptotic formula, and the probabilistic refinement of the fractional clique cover number was studied in relation to the graph entropy in [27].

### 5.2. An Outer Bound via the Asymptotic Spectrum of Graphs

In this subsection, we derive an outer bound for the case when all $\left\{H_{j}\right\}$ are the same, namely, $H_{j}=H$ for all $j\in X_{2}$.

**Theorem** **5.** *$C_{ze}\left(\left[G_{1},\ldots ,G_{|X_{1}|};H,\ldots ,H\right]\right)$ is contained in the region*

$$\begin{array}{c} \left\{\left(R_{1},R_{2}\right):R_{1}\geq 0,R_{2}\geq 0,R_{1}+R_{2}\leq \max_{P_{X_{1}}}\sum_{x_{1}\in X_{1}}P_{X_{1}}\left(x_{1}\right)\Theta \left(G_{x_{1}}\right)+\Theta \left(H,P_{X_{1}}\right)\right\}. \end{array}$$

**Proof.**  Suppose that $\left(A,B\right)\subseteq X_{1}^{n}\times X_{2}^{n}$ is a uniquely decodable codebook pair of length *n*. For any $a^{n}\in A$ and $b^{n}\in B$, let $P_{a^{n},b^{n}}$ denote the joint type of the pair $\left(a^{n},b^{n}\right)$ and

$$J^{n}\left(P_{X_{1},X_{2}}\right)≜\left\{\left(a^{n},b^{n}\right):a^{n}\in A,b^{n}\in B,P_{a^{n},b^{n}}=P_{X_{1},X_{2}}\right\}.$$

By Lemma 11, there are at most ${\left(n+1\right)}^{|X_{1}\left|\right|X_{2}|}$ different joint types over (A,B). Thus by the pigeonhole principle, there exists one joint type $P_{X_{1},X_{2}}^{*}$ such that:

(50) $$|J^{n}\left(P_{X_{1},X_{2}}^{*}\right)|\geq \frac{\left|A||B\right|}{{\left(n+1\right)}^{|X_{1}\left|\right|X_{2}|}}.$$

Notice that for each $\left(a^{n},b^{n}\right)\in J^{n}\left(P_{X_{1},X_{2}}^{*}\right)$, we have:

$$\begin{array}{cc} P_{a^{n}} & =P_{X_{1}}^{*}=\sum_{x_{2}\in X_{2}}P_{X_{1},X_{2}=x_{2}}^{*},\\ P_{b^{n}} & =P_{X_{2}}^{*}=\sum_{x_{1}\in X_{1}}P_{X_{1}=x_{1},X_{2}}^{*}. \end{array}$$

Now we are going to upper bound the cardinality of $J^{n}\left(P_{X_{1},X_{2}}^{*}\right)$. Let $A^{*}$ (resp. $B^{*}$) denote the collection of $a^{n}\in A$ (resp. $b^{n}\in B$) that appears in $J^{n}\left(P_{X_{1},X_{2}}^{*}\right)$, that is, there exists $b^{n}\in B$ (resp. $a^{n}\in A$) such that $P_{a^{n},b^{n}}=P_{X_{1},X_{2}}^{*}$. Then we immediately have

(51) $$|J^{n}\left(P_{X_{1},X_{2}}^{*}\right)\left|\leq \right|A^{*}\left|\right|B^{*}|.$$

Let us now turn to upper bound the cardinalities of $A^{*}$ and $B^{*}$. Since (A,B) is uniquely decodable, by Proposition 1, for any $a^{n}\in A^{*}$ it must hold that $B^{*}$ is an independent set of $G_{a_{1}}⊠G_{a_{2}}⊠\cdots ⊠G_{a_{n}}$. Accordingly,

(52) $$\begin{array}{cc} |B^{*}|\; & \leq \alpha \left(G_{1}^{nP_{X_{1}}^{*}\left(1\right)}⊠G_{2}^{nP_{X_{1}}^{*}\left(2\right)}⊠\cdots ⊠G_{|X_{1}|}^{nP_{X_{1}}^{*}\left(\right|X_{1}\left|\right)}\right). \end{array}$$

Also, for $b^{n}\in B^{*}$, we notice that $A^{*}$ is an independent set of $H^{n}$ with type $P_{X_{1}}^{*}$. To be precise, we have:

(53) $$\begin{array}{cc} |A^{*}|\; & \leq \alpha \left(H^{n}\left[P_{X_{1}}^{*}\right]\right). \end{array}$$

Therefore we have:

$$\begin{array}{cc} \; & \underset{n\to \infty }{lim sup}\frac{1}{n}\log|A||B| \end{array}$$

(54) $$\begin{array}{cc} \; & \leq \underset{n\to \infty }{lim sup}\frac{1}{n}\log\left({\left(n+1\right)}^{|X_{1}\left|\right|X_{2}|}\left|J^{n}\left(P_{X_{1},X_{2}}^{*}\right)\right|\right) \end{array}$$

(55) $$\begin{array}{cc} \; & =\underset{n\to \infty }{lim sup}\frac{1}{n}\log\left|J^{n}\left(P_{X_{1},X_{2}}^{*}\right)\right| \end{array}$$

(56) $$\begin{array}{cc} \; & \leq \underset{n\to \infty }{lim sup}\frac{1}{n}\log|A^{*}\left|\right|B^{*}| \end{array}$$

(57) $$\begin{array}{cc} \; & \leq \underset{n\to \infty }{lim sup}\frac{1}{n}\left[\log\left(\alpha \left(G_{1}^{nP_{X_{1}}^{*}\left(1\right)}⊠\cdots ⊠G_{|X_{1}|}^{nP_{X_{1}}^{*}\left(\right|X_{1}\left|\right)}\right)\right)+\log\left(\alpha \left(H^{n}\left[P_{X_{1}}^{*}\right]\right)\right)\right] \end{array}$$

(58) $$\begin{array}{cc} \; & \leq \underset{n\to \infty }{lim sup}\min_{\eta ,\eta^{\prime }\in \Delta \left(G\right)}\frac{1}{n}\left[\log\left(\eta \left(G_{1}^{nP_{X_{1}}^{*}\left(1\right)}⊠\cdots ⊠G_{|X_{1}|}^{nP_{X_{1}}^{*}\left(\right|X_{1}\left|\right)}\right)\right)+\log\left(\eta^{\prime }\left(H^{n}\left[P_{X_{1}}^{*}\right]\right)\right)\right] \end{array}$$

(59) $$\begin{array}{cc} \; & \leq \underset{n\to \infty }{lim sup}\min_{\eta ,\eta^{\prime }\in \Delta \left(G\right)}\frac{1}{n}\left[\sum_{x_{1}\in X_{1}}nP_{X_{1}}^{*}\left(x_{1}\right)\log\left(\eta \left(G_{x_{1}}\right)\right)+\log\left(\eta^{\prime }\left(H^{n}\left[P_{X_{1}}^{*}\right]\right)\right)\right] \end{array}$$

(60) $$\begin{array}{cc} \; & \leq \max_{P_{X_{1}}}\sum_{x_{1}\in X_{1}}P_{X_{1}}\left(x_{1}\right)\Theta \left(G_{x_{1}}\right)+\Theta \left(H,P_{X_{1}}\right), \end{array}$$

where (54) follows from (50); (55) follows from the fact that $|X_{1}|$, $|X_{2}|$ are fixed when *n* tends to infinity; (56) follows from (51); (57) follows from (52) and (53); (58) follows from Theorem 1 that $\alpha \left(G\right)\leq \min_{\eta \in \Delta (G)}\eta \left(G\right)$ for any graph *G*; (59) follows from Theorem 1 that each η∈Δ(G) is multiplicative with respect to the strong product; and (60) follows from Theorem 1 and Lemma 13.

This completes the proof. □

In particular, considering the DM-TWC such that $|X_{1}|=2$, $H={\bar{K}}_{2}$, $G_{1}={\bar{K}}_{|X_{2}|}$ and $G_{2}=G$ is a general graph, we have the following result.

**Theorem** **6.** *$C_{ze}\left(\left[{\bar{K}}_{|X_{2}|},G;{\bar{K}}_{2},\ldots ,{\bar{K}}_{2}\right]\right)$ is contained in the region*

$$\begin{array}{c} \left\{\left(R_{1},R_{2}\right):R_{1}\geq 0,R_{2}\geq 0,R_{1}+R_{2}\leq \log\left(|X_{2}|+2^{\Theta (G)}\right)\right\}. \end{array}$$

**Proof.**  Recall that: $\Theta \left({\bar{K}}_{n}\right)=\log\left(n\right)$ and $\Theta \left({\bar{K}}_{n},P\right)=H\left(X\right)$. According to Theorem 5, we have:

$$\begin{array}{cc} R_{1}+R_{2}\; & \leq \max_{P_{X_{1}}}\sum_{x_{1}\in X_{1}}P_{X_{1}}\left(x_{1}\right)\Theta \left(G_{x_{1}}\right)+\Theta \left({\bar{K}}_{2},P_{X_{1}}\right)\\ \; & =\max_{P_{X_{1}}}\left\{P_{X_{1}}\left(1\right)\cdot \log\left|X_{2}\right|+P_{X_{1}}\left(2\right)\cdot \Theta \left(G\right)+H\left(X_{1}\right)\right\}\\ \; & =\log\left(|X_{2}|+2^{\Theta (G)}\right), \end{array}$$

where the last equality is achieved by taking $P_{X_{1}}\left(1\right)=\frac{|X_{2}|}{|X_{2}|+2^{\Theta (G)}}$ and $P_{X_{1}}\left(2\right)=\frac{2^{\Theta (G)}}{|X_{2}|+2^{\Theta (G)}}$. □

We remark that Theorem 6 (hence also Theorem 5) could outperform Theorem 2, see the following example.

**Example** **3.** Consider the channel $\left[{\bar{K}}_{5},C_{5};{\bar{K}}_{2},\ldots ,{\bar{K}}_{2}\right]$ where $C_{5}$ is the Pentagon graph. It is well known from [2,12] that $\Theta \left(C_{5}\right)=\frac{1}{2}\log5$. Then by Theorem 6 we have an upper bound on the sum-rate $R_{1}+R_{2}\leq \log\left(5+\sqrt{5}\right)\approx 2.8552$, while Theorem 2 only gives an upper bound $R_{1}+R_{2}\leq 2.9069$.

### 5.3. Explicit Constructions

In this subsection, we present explicit constructions of uniquely decodable codebook pairs which could attain the outer bound of Theorem 6 in certain special cases.

**Theorem** **7.** *Let m be a prime power, $|X_{2}|=q=ms$ and $G=K_{m}⊔\cdots ⊔K_{m}$ be a disjoint union of s cliques. Then $C_{ze}^{sum}\left([{\bar{K}}_{q},G;{\bar{K}}_{2},\ldots ,{\bar{K}}_{2}]\right)=\log\left(q+s\right)$.*

**Proof.**  First by Theorem 6, we have an upper bound on the sum-capacity given by

(61) $$C_{ze}^{sum}\left([{\bar{K}}_{q},G;{\bar{K}}_{2},\ldots ,{\bar{K}}_{2}]\right)\leq \log\left(|X_{2}|+2^{\Theta (G)}\right)=\log\left(q+s\right).$$

Next, we consider the lower bound. Notice that $G=K_{m}⊠{\bar{K}}_{s}$. We can reformulate the channel accordingly as:

$$\left[{\bar{K}}_{q},G;{\bar{K}}_{2},\ldots ,{\bar{K}}_{2}\right]=\left[{\bar{K}}_{m},K_{m};{\bar{K}}_{2},\ldots ,{\bar{K}}_{2}\right]⊠\left[{\bar{K}}_{s};K_{1},\ldots ,K_{1}\right],$$

where the first $\left[{\bar{K}}_{m},K_{m};{\bar{K}}_{2},\ldots ,{\bar{K}}_{2}\right]$ corresponds to a channel with input alphabets $X_{1}^{\left(1\right)}=\left\{1,2\right\}$ and $X_{2}^{\left(1\right)}=\left\{1,\ldots ,m\right\}$; and the second $\left[{\bar{K}}_{s};K_{1},\ldots ,K_{1}\right]$ is with input alphabets $X_{1}^{\left(2\right)}=\left\{1\right\}$ and $X_{2}^{\left(2\right)}=\left\{1,\ldots ,s\right\}$. Together with Lemma 1, we have:

(62) $$C_{ze}^{sum}\left([{\bar{K}}_{q},G;{\bar{K}}_{2},\ldots ,{\bar{K}}_{2}]\right)\geq C_{ze}^{sum}\left([{\bar{K}}_{m},K_{m};{\bar{K}}_{2},\ldots ,{\bar{K}}_{2}]\right)+C_{ze}^{sum}\left([{\bar{K}}_{s};K_{1},\ldots ,K_{1}]\right).$$

On the one hand, it is easy to see that:

(63) $$C_{ze}^{sum}\left([{\bar{K}}_{s};K_{1},\ldots ,K_{1}]\right)=\log s$$

since this is a clean channel and Alice and Bob could always communicate without error. On the other hand, by Lemma 10, we obtain:

(64) $$C_{ze}^{sum}\left([{\bar{K}}_{m},K_{m};{\bar{K}}_{2},\ldots ,{\bar{K}}_{2}]\right)\geq \log\left(m+1\right).$$

In fact, letting q=m, $q^{\prime }=2$ and τ=1 in Lemma 10, we have a pair (C,Y(C)) satisfying that C is an *m*-ary [n,k] linear code and $Y\left(C\right)\subseteq F_{2}^{n}$ such that

(65) |Y(C)|≥nk∏i=1∞(1−m−i).

Now let A=Y(C) and B=C, then it is easy to see that (A,B) is a uniquely decodable codebook pair with respect to the channel $\left[{\bar{K}}_{m},K_{m};{\bar{K}}_{2},\ldots ,{\bar{K}}_{2}\right]$. The corresponding sum-rate is

limn→∞1nlog|A||B|=limn→∞1nlogmk+lognk∏i=1∞(1−m−i)=limn→∞knlogm+hkn.

Taking k/n=m/(m+1), we obtain a lower bound log(m+1) on the best possible sum-rate, that is, (64).

Combining (62)–(64), we have $C_{ze}^{sum}\left([{\bar{K}}_{q},G;{\bar{K}}_{2},\ldots ,{\bar{K}}_{2}]\right)\geq \log\left(m+1\right)+\log s=\log\left(q+s\right)$, which also implies an explicit uniquely decodable codebook pair for the channel $\left[{\bar{K}}_{q},G;{\bar{K}}_{2},\ldots ,{\bar{K}}_{2}\right]$ based on the argument of Lemma 1. Then, together with (61), the proof is complete. □

## 6. Concluding Remarks

In this paper, we investigated the non-adaptive zero-error capacity region of the DM-TWC and provided several single-letter inner and outer bounds, some of which coincide in certain special cases. Determining the exact zero-error capacity region of a general DM-TWC remains an open problem, and clearly a difficult one, since it includes the notorious Shannon capacity of a graph as a special case. Despite this inherent difficulty, the problem is richer than the graph capacity setting, and we believe it deserves further study in order to obtain tighter bounds and smarter constructions.

One appealing direction is to extend the Lovász’s semi-definite relaxation approach in order to obtain tighter outer bounds, mimicking the graph capacity case. This, however, does not seem to be a simple task. In particular, one may ask whether the natural quantity $\rho (\left\{G_{i}\right\},\left\{H_{j}\right\})$ defined in (2), which upper-bounds the one-shot zero-error sum-capacity, is sub-multiplicative with respect to the graph strong product, in which case it would also serve as an upper bound for the zero-error sum-capacity. This is however not evident, in part since the problem (2) is not a semi-definite program. We have also considered other variations of the program (2). In particular, we have attempted to modify the non-linear constraints $\langle E_{i,j},\Gamma \rangle \langle E_{i,m},\Gamma \rangle =0$ to be of a linear form 〈A,Γ〉=0 for some suitable symmetric matrix *A*. We have also looked at some variants of the orthonormal representation. For example, we considered the case where each graph vertex *a* is labelled by a unit vector $v_{a}$, and if two vertices *a* and $a^{\prime }$ are nonadjacent $a\overset{b}{≁}a^{\prime }$ if and only if b∈F for some set *F*, then the vector projections of $v_{a}$ and $v_{a^{\prime }}$ onto the subspace spanned by the vectors in *F* are orthogonal. However, proving sub-multiplicativity in any of these settings has so far resisted our best efforts.

It would be also of much interest to consider the adaptive zero-error capacity of the DM-TWC. Allowing Alice and Bob to adapt their transmissions on the fly can in general enlarge the zero-error capacity region. As a simple example, note that a point-to-point channel with noiseless feedback is a special case of the DM-TWC (where Bob has no information to send). In [2], Shannon explicitly derived the zero-error capacity with feedback for the point-to-point channel, and pointed out that for the channel corresponding to Pentagon graph this capacity is given by log(5/2)≈1.32. This is strictly larger than the zero-error capacity without feedback (log5)/2≈1.16, which can be thought of in this case as the non-adaptive zero-error capacity of the channel. Exploring the differences between the adaptive and non-adaptive zero-error capacity regions of a general DM-TWC remains a challenging future work.

## Data Availability

Not applicable.
